# Facile Fabrication of Protein–Macrocycle Frameworks

**DOI:** 10.1021/jacs.0c10697

**Published:** 2021-01-20

**Authors:** Kiefer O. Ramberg, Sylvain Engilberge, Tomasz Skorek, Peter B. Crowley

**Affiliations:** † School of Chemistry, National University of Ireland Galway, University Road, Galway, H91 TK33, Ireland; ‡ Swiss Light Source, Paul Scherrer Institut, Villigen PSI, 5232, Switzerland

## Abstract

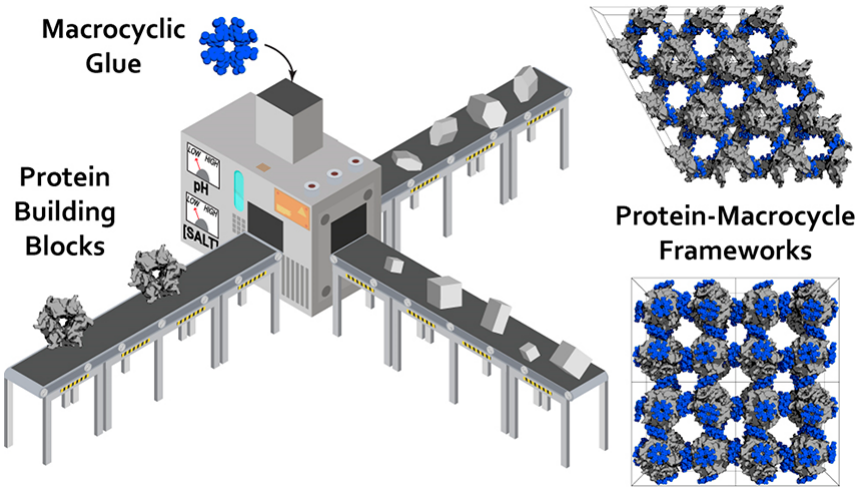

Precisely defined protein aggregates, as exemplified by crystals, have applications in functional materials. Consequently, engineered protein assembly is a rapidly growing field. Anionic calix[n]arenes are useful scaffolds that can mold to cationic proteins and induce oligomerization and assembly. Here, we describe protein-calixarene composites obtained via cocrystallization of commercially available sulfonato-calix[8]arene (**sclx**_**8**_) with the symmetric and “neutral” protein RSL. Cocrystallization occurred across a wide range of conditions and protein charge states, from pH 2.2–9.5, resulting in three crystal forms. Cationization of the protein surface at pH ∼ 4 drives calixarene complexation and yielded two types of porous frameworks with pore diameters >3 nm. Both types of framework provide evidence of protein encapsulation by the calixarene. Calixarene-masked proteins act as nodes within the frameworks, displaying octahedral-type coordination in one case. The other framework formed millimeter-scale crystals within hours, without the need for precipitants or specialized equipment. NMR experiments revealed macrocycle**-**modulated side chain p*K*_a_ values and suggested a mechanism for pH-triggered assembly. The same low pH framework was generated at high pH with a permanently cationic arginine-enriched RSL variant. Finally, in addition to protein framework fabrication, **sclx**_**8**_ enables *de novo* structure determination.

## Introduction

Protein-based materials have great potential to serve society.^1−4^ With their periodic arrangement of functional building blocks, crystals have applications in catalytic devices.^3−7^ Porous crystals are of particular interest considering their capacity to capture (store) and transform biomolecules.^6,8−14^ While great advances have been achieved with metal organic frameworks (MOFs)^12^ and covalent organic frameworks (COFs),^13^ protein-based frameworks have proved more challenging.^11,14−17^ Yet, biocompatible and biodegradable frameworks are highly desirable given the demands for new therapeutics and biomaterials as well as sustainable manufacturing processes.^2^ This paper describes the facile fabrication of millimeter-scale, high-porosity, solid-state composites of precisely arrayed protein and synthetic components.

Designed protein oligomerization and protein crystal engineering are progressing toward the goal of protein-based devices.^9−11,14−28^ The application of Coulombic forces for guided assembly continues to deliver satisfactory results such as the cocrystallization of binary mixtures of oppositely charged homologues.^25−27^ Multivalent ligands and “molecular glues” offer alternative approaches to controlled assembly without the requirement for engineered surface features in the target protein.^3,18−23^ For example, anionic calix[n]arenes that host arginine or lysine side chains^29−32^ can direct the assembly of cationic proteins.^20,33−37^ The commercially available sulfonato-calix[8]arene (**sclx**_**8**_), a 1.5 kDa flexible phenolic macrocycle with variable cavity dimensions,^29^ a solvent accessible surface area (ASA) of ∼1600 Å^2^, and a formal net charge ranging from −8 to −12,^38^ shows particular promise.^35−37^ We have demonstrated autoregulated oligomerization of the lysine-rich cytochrome *c* (cyt*c*, isoelectric point, p*I* ∼ 9.5), with 1 equiv **sclx**_**8**_ forming a tetramer, and 3 equiv yielding a calixarene-coated (encapsulated) protein.^35^ The ligand:protein ratio influences also the formation of cyt*c*-**sclx**_**8**_ crystalline frameworks with varying porosities, ranging from 65 to 85% solvent content.^35,37^ The most porous framework is mediated exclusively by protein-calixarene contacts and requires at least 3 equiv **sclx**_**8**_ with respect to the cationic protein. Crystal engineering and the use of effector ligands have provided access to different architectures including a duplicated framework.^37^ Here, we describe the preparation of crystalline frameworks comprising a symmetric “neutral” protein and **sclx**_**8**_.

The 6-bladed β-propeller *Ralstonia solanacearum* lectin (RSL, p*I* ∼ 6.5) was selected as the model protein.^39−43^ RSL is a rigid, *C*_3_-symmetric spheroid with high thermal stability making it an interesting candidate for protein-based frameworks.^43^ Trimeric RSL possesses pseudo *C*_6_-symmetry due to the ∼40% sequence identity between its N- and C-terminal halves. Such high symmetry is advantageous for framework fabrication as evidenced with other lectins,^19,23,44^ related β-propellers,^45^ ferritin,^15,16,24,25^ viral capsids,^8,46^ and engineered cages.^6,28,47−49^**sclx**_**8**_-mediated assembly was tested with RSL and several variants including RSLex^43^ and MK-RSL (each containing one extra lysine), and two arginine-enriched mutants, RSL-R_6_ and RSL-R_8_ (Figures S1 and S2). In RSL-R_6_, the three lysines of native RSL are replaced by arginine.^42^ RSL-R_8_ includes these mutations as well as two acidic residues replaced by arginine. The chemically modified variants, methylated RSL (RSL*) and acetylated RSL (RSL-Ac), were tested also.

We present three types of RSL-**sclx**_**8**_ frameworks dependent on the protein charge characteristics (pH trigger) and the cocrystallization conditions. Two of the frameworks require acidic conditions and are porous with >55% solvent content and pore diameters >3 nm. These frameworks are consistent with protein encapsulation^50^ by calix[8]arene in solution^35^ and suggest a molecular basis for reentrant condensation.^51,52^ The low pH framework was recapitulated with the highly cationic RSL-R_8_ variant. NMR experiments provide further evidence of a pH trigger, arising from protonation of acidic side chains in RSL. One of the fabrication processes is rapid (hours), yields millimeter-scale crystals, and requires neither precipitants nor specialized equipment. Thus, we demonstrate the general utility of **sclx**_**8**_ for protein framework assembly as well as X-ray structure determination by anomalous methods.

## Experimental Section

### Materials

Stock solutions of **sclx**_**8**_ (Tokyo Chemical Industry) were prepared in water, and the pH was adjusted to 8.0.

### Protein Production

Unlabeled and ^15^N-labeled RSL samples were produced in *E. coli* BL21 transformed with the pET25rsl plasmid. The modified pET25rsl vectors that encode RSL-R_6_ and RSLex were reported previously.^42,43^ The vectors encoding RSL-R_8_ (K25R/K34R/E43R/D46R/K83R) and MK-RSL were produced by Genscript. All proteins, except RSL-R_8_, were purified by mannose-affinity chromatography.^39^ Attempts to purify RSL-R_8_ by affinity chromatography failed due to the coelution of *E. coli* proteins, likely as a consequence of arginine “stickiness”.^53^ Consequently, RSL-R_8_ was purified on carboxymethyl resin equilibrated with 0.02 M potassium phosphate and 0.2 M NaCl at pH 6.0, and eluted with the same buffer plus 1 M NaCl. Methylation and acetylation of RSL were performed as described.^42,43^ Mass analysis of RSL-R_8_ and MK-RSL was performed with an Agilent 6460 Triple Quadrupole LC/MS (Figure S3, Table S1). Protein concentrations were determined spectrophotometrically with ε_280_ = 44.46 mM^–1^cm^–1^ for the monomer.

### Cocrystallization Trials

All experiments were performed with d-fructose bound RSL and variants. Protein-**sclx**_**8**_ cocrystals were obtained at 20 °C by using commercial (JCSG++ HTS, Jena Bioscience) or homemade screens, applied with an Oryx 8 robot (Douglas Instruments). Generally, the crystals were reproduced manually by hanging-drop vapor diffusion in 24 well Greiner plates. Protein concentrations ranged from 0.8 to 1.8 mM. Crystals were obtained in JCSG++ HTS conditions **B1** (0.8 M ammonium sulfate and 0.1 M sodium citrate pH 4.0), **C11** (2.0 M ammonium sulfate and 0.1 M sodium acetate pH 4.6), **E2** (2.0 M ammonium sulfate, 0.2 M sodium chloride and 0.1 M MES pH 6.5), and **G11** (2.0 M ammonium sulfate and 0.1 M BIS-TRIS pH 5.5). Homemade screens included 0.8–2.4 M (NH_4_)_2_SO_4_, 0.1 M buffer, and 0 or 0.2 M NaCl, (Li)_2_SO_4_ or MgCl_2_. The buffers tested (pH values indicated in parentheses, not corrected for the presence of salts) were glycine-HCl (2.2), citrate (4.0), acetate (4.6), MES (6.8), Tris-HCl (8.5), or CAPS (9.5). Typically, screens included 0, 1, 2, 4, 8, 16, 32, or 64 mM **sclx**_**8**_. In the case of RSL-R_8_, the Jena screen was tested at 50 and 100 mM **sclx**_**8**_. Crystals were obtained also by incubation of protein–ligand mixture in microcentrifuge tubes. In this simplified batch crystallization, protein-**sclx**_**8**_ mixtures were prepared in 20 mM acetate or phosphate, 50 mM NaCl, pH adjusted to 4.0, and incubated at 4 °C. Crystallization drops were imaged using an Olympus SZX16 stereomicroscope and a Olympus DP25 digital camera.

### X-ray Data Collection, Processing, and Model Building

Crystals were cryo-protected in the crystallization solution supplemented with 20–25% glycerol and cryo-cooled in liquid nitrogen. Diffraction data were collected at beamline PROXIMA-2A, SOLEIL synchrotron (Saint-Aubin, France) with an Eiger X 9M detector (Tables S2–S4). Data were processed using the autoPROC pipeline.^54^ Data were integrated in XDS^55^ and the integrated intensities were scaled and merged in AIMLESS^56^ and POINTLESS^57^ in CCP4 and assessed for pathologies in phenix.Xtriage.^58^ Structures were solved by molecular replacement in PHASER,^59^ using the RSL monomer (derived from PDB 2BT9(39)) as a search model. For *de novo* phasing experiments data were collected at beamline X06DA, Swiss Light Source (Villigen, Switzerland). To maximize anomalous scattering from sulfur atoms, diffraction data were collected at a wavelength of 2.07 Å (Table S3). A single data set collected at this energy sufficed for *de novo* structure determination.^60^ Diffraction frames (deposited on Zenodo, DOI: 10.5281/zenodo.3944486) were integrated using XDS and scaled with AIMLESS and POINTLESS. Substructure determination, phasing, and model building were performed in SHELX.^61^ A second high-resolution data set used for refinement was collected on the same crystal at a wavelength of 0.97 Å and processed using the autoPROC pipeline (Table S3). The coordinates for **sclx**_**8**_ (PDB id EVB) and d-fructose (PDB id BDF) were added to each model. Iterative cycles of model building in COOT^62^ and refinement in phenix.refine^58^ were performed until no further improvements in the R_free_ or electron density were obtained. All of the structures were validated in MolProbity.^63^ High resolution refined coordinates and structure factors were deposited in the Protein Data Bank (Tables 1 and S2–S4). Protein–ligand interface areas were calculated in PDBe PISA.^64^ Crystal porosity was analyzed in MAP_CHANNELS.^65^ The molar protein concentration, [P], in each crystal form was calculated by
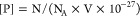
where N is the number of molecules of P in the unit cell, N_A_ is Avogadro’s number, and V is the unit cell volume (Å^3^).

**Table 1:** Cocrystallization Conditions and Structure Properties

**Form**	**Protein**	**equiv sclx**_**8**_	**(NH**_**4**_**)**_**2**_**SO**_**4**_**(M)**	**Buffer**	**pH**	**Additive (0.2 M)**	**Space Group**	***a*****x*****b*****x*****c*****(Å)**	**Res (Å)**	**PDB id**
I	RSL	80	1.6	CAPS	9.5	Li_2_SO_4_	*P*2_1_3	64^3^	1.2	6Z60
	RSL	80	1.6	Tris-HCl	8.5	Li_2_SO_4_			1.3	6Z62
	RSL-R_6_	50	1.6	Tris-HCl	8.5	Li_2_SO_4_		(3)	1.1	6Z5Z
	RSL	50	2.4	MES	6.8				1.1	6Z5W
	RSL	80	2.0	acetate	4.8				1.1	6Z5X
										
II	RSL	50	0.8	citrate	4.0		*I*23	104^3^	1.3	6Z5G
	RSL	10	0.8	Gly-HCl	2.2	MgCl_2_			1.6	6Z5M
										
III	RSL	10		acetate	4.0		*P*3	60 × 60 × 64	1.3	6Z5Q
	RSL*	10		acetate	4.0			60 × 60 × 64	1.3	7ALF
	RSLex	15		acetate	4.0			60 × 60 × 64	1.5	7ALG
	RSL-R_8_	50	1.3	Tris-HCl	8.5	Li_2_SO_4_		60^3^	1.4	6Z5P

### NMR Characterization

Samples typically comprised 0.1–1.0 mM ^15^N-labeled protein in 20 mM phosphate buffer, 50 mM NaCl, 5 mM d-fructose, and 10% D_2_O. Samples in 20 mM acetate buffer (instead of phosphate) were tested also. Ligand titrations were performed with μL aliquot additions of 0.1 M **sclx**_**8**_. 2D ^1^H–^15^N HSQC watergate spectra were acquired at 30 °C with 4 or 8 scans and 64 increments on a Varian 600 MHz spectrometer with a HCN cold probe. Data processing and analysis were performed in NMRpipe^66^ and CCPN,^67^ respectively. Binding isotherms were obtained by plotting the chemical shift perturbation (Δδ) as a function of the **sclx**_**8**_ concentration. Nonlinear least-squares fits to a one-site binding model were performed, with Δδ and [**sclx**_**8**_] as the dependent and independent variables, respectively, and the dissociation constant (*K*_d_) and maximum chemical shift change (Δδ_max_) as the fit parameters. pH titration curves were generated from ^1^H–^15^N HSQC spectra of RSL, in the presence of 0 or 5 mM **sclx**_**8**_, pH adjusted in increments of 0.2 pH units. The pH was measured before and after each HSQC data acquisition. The pH dependence of the chemical shifts was analyzed using nonlinear least-squares fits of the data to the modified Henderson–Hasselbalch equation
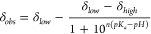
where p*K*_*a*_ is the ionization constant, δ_low_ and δ_high_ are the low and high pH chemical shift plateaus, and *n* is the apparent Hill coefficient.^68^

### Empirical p*K*_a_ Calculations

The p*K*_a_ values of the acidic residues measured by NMR were compared with empirical estimates obtained by using PROPKA3.2.^69^ In this version of the p*K*_a_ predictor, the parameter sets account for noncovalent interactions with ligand groups. The PROPKA3.2 calculations were performed with the coordinates of the *P*3 RSL-**sclx**_**8**_ crystal structure (PDB 6ZQ5) with and without the **sclx**_**8**_ coordinates included in the model.

## Results

### RSL-**sclx**_**8**_ Cocrystal Forms

Despite its “neutral” character, RSL cocrystallized with the highly anionic **sclx**_**8**_ under a broad variety of conditions (Table 1 and Figure S4). Data collection at SOLEIL synchrotron revealed three distinct crystal forms with high-quality diffraction properties (Tables 1, 2, and S2–S4).

**Table 2:** RSL-**sclx**_**8**_ Cocrystal Forms and Properties

**Form**	**Space Group**	**RSL:sclx**_**8**_^a^	**[P] (mM)**^b^	**S.C. (%)**^c^	**Pore** Ø **(nm)**^d^
I	*P*2_1_3	1:1	76	36	1.7
II	*I*23	1:2	36	66	4.2
III	*P*3	1:1	50	59	2.8

a Protein:ligand ratio per RSL monomer.

b Calculated protein concentration based on unit cell contents.

c Solvent content estimated from total mass (protein plus **sclx**_**8**_).

d Diameter of widest pore, calculated in MAP_CHANNELS.^65^

Sitting-drop vapor-diffusion experiments with a commercial screen yielded RSL-**sclx**_**8**_ cocrystals in 2.0 M ammonium sulfate with different buffers (see Experimental Section). This crystal form (I) grew at 40–80 equiv **sclx**_**8**_ over 7–10 days. Homemade screens extended these results to 1.6–2.4 M ammonium sulfate and buffers ranging from acetate pH 4.8 to CAPS pH 9.5 (Table 1). The growth of crystal form I was independent of the pH, occurring in acidic or basic conditions where the protein is cationic or anionic, respectively. This observation, together with the high ammonium sulfate concentration (Debye screening), suggests that attractive charge–charge interactions have a minor contribution to this cocrystallization process. While **sclx**_**8**_ is always anionic, RSL is cationic or anionic at low or high pH, respectively. Therefore, attractive charge–charge interactions between the protein and calixarene occur only at pH ≤ 5.

**Figure 1:**
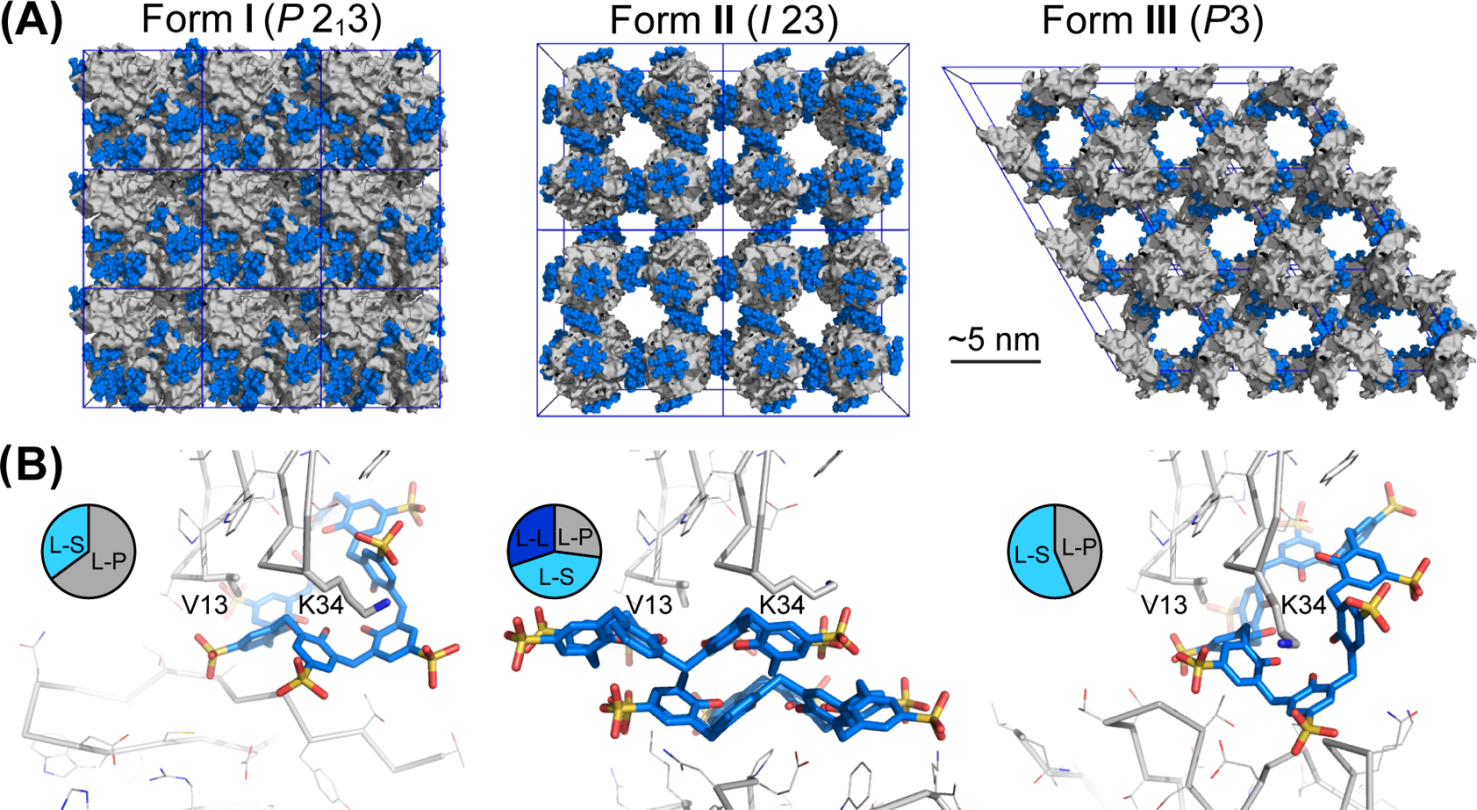
(A) Crystal packing in RSL-**sclx_8_**cocrystal forms I (*P*2_1_3), II (*I*23), and III (*P*3). Note the high porosity of the *I*23 and *P*3 forms, with nanometer-scale solvent channels. Proteins shown as gray surfaces, and **sclx**_**8**_ shown as blue spheres. (B) Details of the principal protein-**sclx_8_**-protein interfaces in each crystal form, with RSL shown as the monomer for clarity. The Val13 and Lys34 binding patch is common to each crystal form. Pie charts show area proportions of **sclx_8_**-mediated interfaces (see Table S4). L, ligand; P, protein; S, solvent.

**Figure 2:**
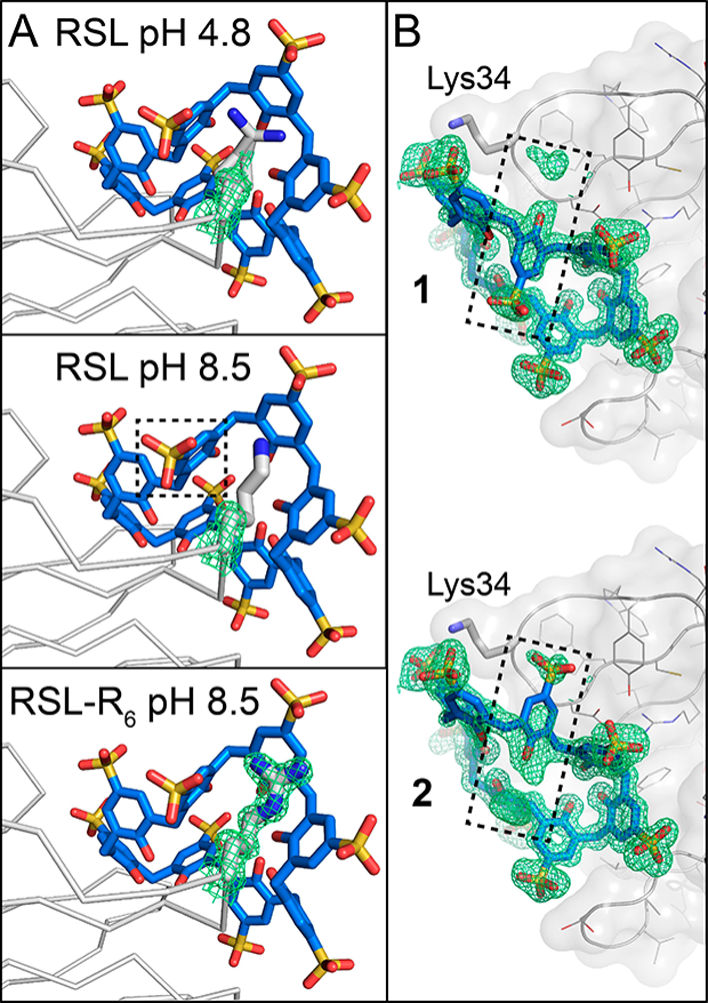
(A) Disorder at the Lys34-**sclx**_**8**_ site increases with increasing pH in the *P*2_1_3 form. In contrast, Arg34 is fully defined in the RSL-R_6_ variant. (B) Electron density in the RSL-**sclx**_**8**_ crystal structure at pH 8.5 is consistent with alternate conformations for one calixarene monomer, highlighted with a dashed box. Alternate conformers 1 and 2 of **sclx**_**8**_ were modeled at 55 and 45% occupancy, respectively. 2Fo-Fc electron density maps (contoured at 1.0 σ) are shown as green mesh.

Crystal form I was solved with an asymmetric unit comprising one RSL monomer and one **sclx**_**8**_ in the cubic space group *P2*_1_3. An essentially identical structure was obtained with RSL-R_6_. This tightly packed crystal form with 36% solvent content (Figure 1 and Table 2) involves two protein–protein crystal contacts and five protein-calixarene interfaces. The protein–protein interfaces bury 100 or 140 Å^2^ per molecule and are typical crystal contacts. The protein-**sclx**_**8**_ interfaces bury ∼65% of the calixarene, while the remainder is solvent exposed. **sclx**_**8**_ adopts a highly puckered conformation and molds neatly to one RSL monomer (Figures 1B and 2A), burying 585 Å^2^ and entrapping adjacent residues Val13 and Lys34 in niches formed by two and three proximal phenolic units, respectively. Another calixarene cavity interacts *exo* to RSL and masks several residues, including Asp32 (*vide infra*) and Tyr37, adjacent to the sugar binding site. The other side of the **sclx**_**8**_ forms an interface that is ∼3-fold smaller (180 Å^2^) and binds Lys83 and Ala85 of a second RSL monomer. Calixarene complexation of two aliphatic side chains (Val13 and Ala85) via CH-π bonds suggests that the hydrophobic effect is important to this crystal form that grows in high salt conditions.

While the hydrophobic effect appears to be important in crystal form I, charge–charge interactions are likely to be inconsequential. Support for this argument arises from the growth of these crystals in both acidic and alkaline conditions (*vide supra*) and from the disorder at the Lys34 site (Figure 2). While the electron density maps clearly indicate an extended conformation for Lys83 across the pH range, Lys34 was modeled in two conformations with high temperature factors^70^ (indicative of flexibility/motion) even in the pH 4.8 structure. The encapsulating portion of **sclx**_**8**_ has correspondingly high temperature factors (∼30 Å^2^) compared to the rest of the ligand (∼10 Å^2^). At pH ≥ 8.5, the Lys34 side chain is completely disordered beyond C^β^ and one monomer of **sclx**_**8**_ flicks between two opposing conformations (Figure 2B). These results suggest that the Lys34 side chain is deprotonated and does not engage **sclx**_**8**_ via the typical salt bridge interactions.^33−37^ The replacement of Lys34 with Arg34 in RSL-R_6_ yields a better-defined interface (Figure 2A). The high p*K*_a_ of Arg^71^ ensures protonation and salt bridge formation with the sulfonic acids. Presumably, the increased bulk of Arg relative to Lys also contributes to stabilize the interface. Another aspect of the disorder around **sclx**_**8**_ concerns the N-terminal Ser1. This protonated residue is disordered despite its location at ∼5 Å from the nearest sulfonic acid, suggesting again that charge–charge interactions are minor in crystal form I.

**Figure 3:**
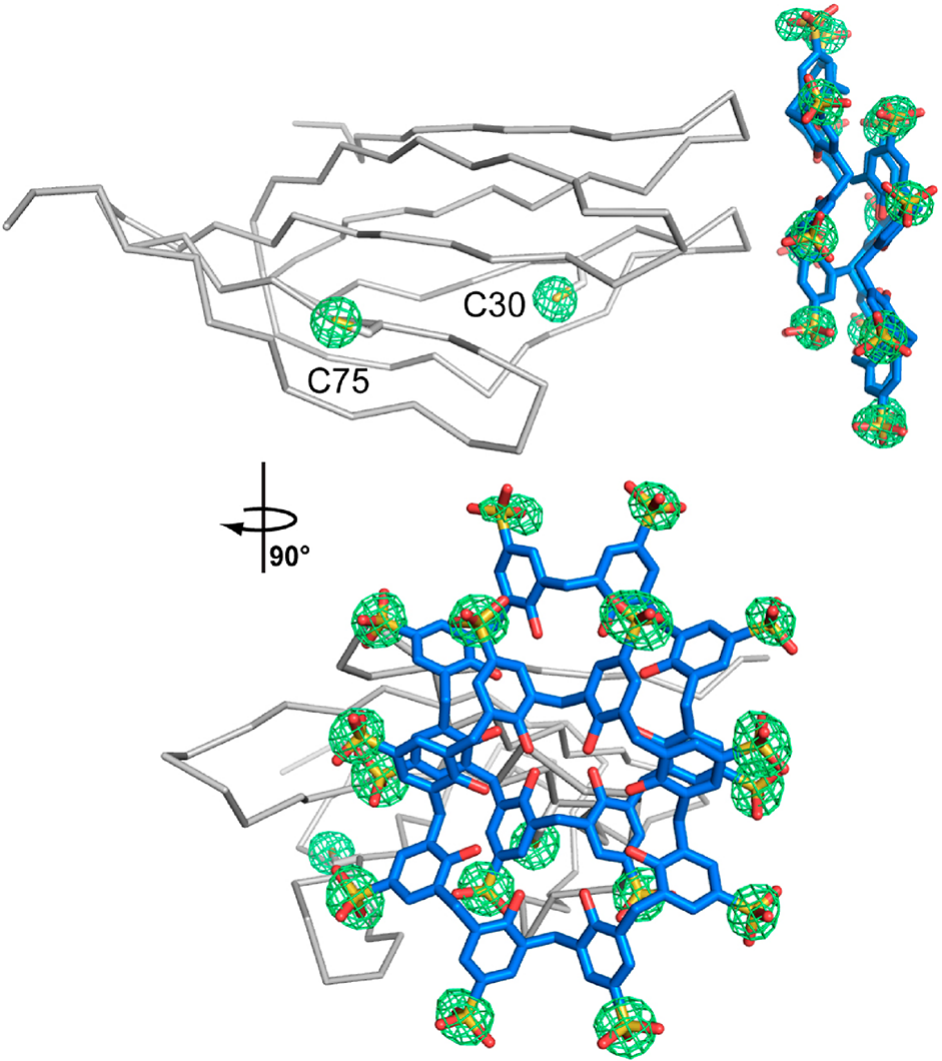
Fourier maps (green mesh) calculated from sulfur anomalous data and contoured at 4.0 σ in crystal form II (*I*23). The RSL monomer is shown as the C^α^ trace, the **sclx**_**8**_ dimer and cysteine side chains are shown as sticks.

The commercial screen also yielded crystals, form II, in 0.8 M ammonium sulfate and citrate pH 4.0. In this case, 10 equiv **sclx**_**8**_ was sufficient and the crystals appeared within 4–5 days (Table 1). Homemade screens including acetate pH 4.0 or glycine-HCl pH 2.2 also yielded crystal form II. The requirement for acidic conditions, in which the protein is cationic, suggests that attractive charge–charge interactions are important for cocrystallization of form II. The structure was solved in the cubic space group *I*23 with an asymmetric unit that contains two molecules of **sclx**_**8**_ per monomer of RSL (Table 2). The unit cell is a remarkable assembly of eight RSL trimers connected by **sclx**_**8**_ dimers (Figure 1). Here, **sclx**_**8**_ occurs in the fully extended, pleated loop conformation (ASA 1600 Å^2^) that presents four shallow cavities on each face. The dimer, composed of two identical **sclx**_**8**_, is a staggered assembly that buries 480 Å^2^ per calixarene and involves multiple CH−π, OH−π, π–π, and anion−π interactions (Figures 3 and S5). Two of the sulfonic acid substituents are encapsulated in shallow cavities of the partner calixarene. This highly anionic species (formal charge exceeding −16) dominates the crystal packing which is a porous assembly devoid of any protein–protein crystal contacts (66% solvent content, pore diameter ∼4 nm, Table 2). Two distinct patches of RSL bind to either side of the **sclx**_**8**_ dimer. The larger protein-**sclx**_**8**_ interface (500 Å^2^) entraps Asn42, Pro44, and Trp74. Each side chain makes van der Waals contact with two phenolic rings and one methylene of **sclx**_**8**_. This cluster of residues is flanked by Lys25 and Lys83, both of which form salt bridges to the calixarene. Glu43 (*vide infra*) is completely masked by the calixarene and forms hydrogen bonds to the lower rim phenols. The smaller protein-**sclx**_**8**_ interface (240 Å^2^) entraps Val13 and Lys34, with features similar to the principal interface in crystal form I (*P2*_1_3). In this case, the electron density was clear for Lys34 and both the side chain and the calixarene were modeled with low temperature factors. Charge interactions appear to be important for crystal form II as the calixarene is present as a dimer, all three lysines of RSL participate in **sclx**_**8**_ complexation, and the crystals grow only at pH ≤ 4.0.

The sulfur content of **sclx**_**8**_ was taken advantage of and the *I*23 structure was solved *de novo* by single-wavelength anomalous dispersion (SAD) of sulfur atoms.^60^ The crystal architecture with two **sclx**_**8**_ per RSL monomer adds 16 sulfur atoms per asymmetric unit in addition to two cysteine residues (Figure 3). *De novo* phasing using the anomalous sulfur signal was straightforward and yielded a perfectly interpretable electron density map suited to completely automated model building (Table S3). These data demonstrate the utility of **sclx**_**8**_ for framework fabrication as well as crystal structure determination.

**Figure 4:**
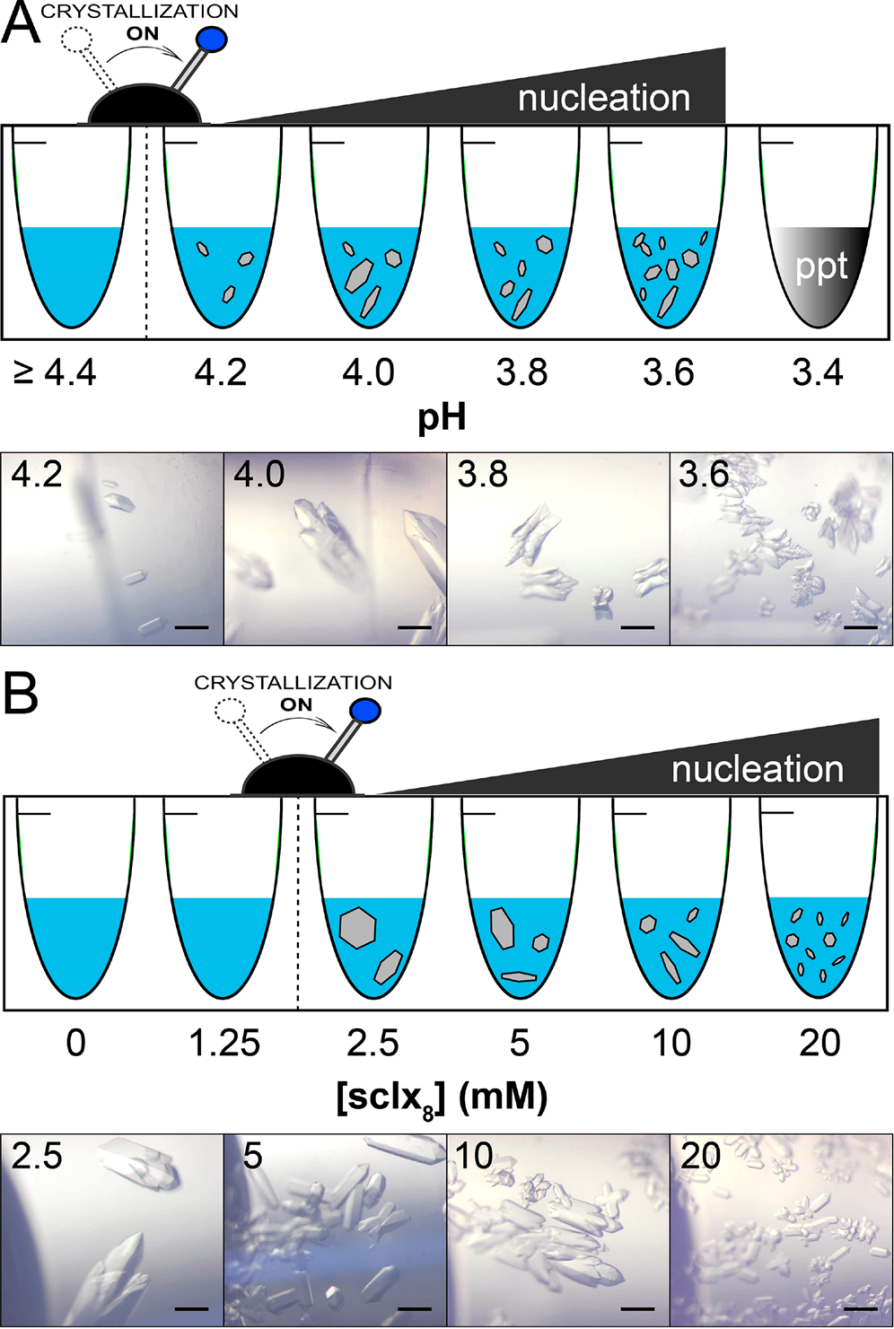
RSL-**sclx**_**8**_ cocrystallization in the absence of precipitant at 4 °C. (A) pH dependence of crystal growth at 1 mM RSL, 10 mM **sclx**_**8**_ in 20 mM phosphate buffer, and 50 mM NaCl. Cocrystallization was triggered at pH ≤ 4.2. Microcrystalline precipitate occurred at pH 3.4 (Figure S5). (B) Cocrystallization dependence on the **sclx**_**8**_ concentration at pH 4.0. Cocrystallization was triggered at **sclx**_**8**_ ≥ 2.5 mM. Microscope images were acquired after 3 h incubation. Scale bars are 200 μm.

Crystal form III was discovered in the course of NMR experiments when overnight sample storage at 4 °C yielded crystals. Form III also requires acidic conditions, and grows rapidly (2–3 h) in the absence of precipitant. Protein–ligand mixtures in 20 mM phosphate or acetate buffer and 50 mM NaCl at pH 4.0, yielded crystals upon incubation at 4 °C (Figures 4, S6–S8). At 10 equiv **sclx**_**8**_, cocrystallization is switched on at pH ≤ 4.2 (Figure 4A). Nucleation increased with decreasing pH and a fine microcrystalline precipitate formed at pH 3.4 (Figure S7). This precipitate was redissolved simply by raising the pH or by increasing the **sclx**_**8**_ concentration (Figure S8), consistent with reentrant condensation.^51,52^ Compared to crystal forms I and II, the growth of form III was more sensitive to the protein:calixarene ratio. At pH 4.0, 2.5 equiv **sclx**_**8**_ was sufficient for cocrystallization. This form was distinguished also by its size, yielding millimeter-scale crystals overnight at low **sclx**_**8**_ concentrations (Figure 4). While the crystal growth was pH sensitive, crystals transferred to buffer at pH 7.4 were stable for 2–3 h. Above pH 7.4, the crystals dissolved within minutes. Considering the low pH and low ionic strength, charge–charge interactions dominate crystal form III, which was reproduced with RSL variants (RSL* and RSLex) under identical conditions, and with the highly cationic RSL-R_8_ at pH 8.5 in ammonium sulfate (Table 1 and Figure S4).

Crystal form III was solved in space group *P*3 with one **sclx**_**8**_ per RSL monomer. Similar to the *I*23 form, protein-calixarene contacts dominate the porous *P*3 assembly (59% solvent content, pore diameter ∼3 nm, Table 2) and there are no protein–protein contacts (Figure 1A). **sclx**_**8**_ adopts a highly puckered conformation, akin to that in form I (*P2*_1_3), but it is substantially more solvent-exposed (Figure 1B and Table S5). Two protein-**sclx**_**8**_ interfaces are involved in this assembly. In the largest interface, 395 Å^2^ of **sclx**_**8**_ is buried by the encapsulation of Val13 and Lys34. Surprisingly, **sclx**_**8**_ also accommodates Asp32 in a shallow cavity formed by two adjacent phenolic units. The Asp32 side chain is rotated slightly (relative to the *P2*_1_3 and *I*23 forms) such that it no longer forms a salt bridge with Arg17, hinting at a change in its protonation state. The second interface buries 260 Å^2^ of the calixarene and involves three short loops of the neighboring RSL monomer pressed up against the calixarene portion that encapsulates Lys34. These loops are capped by Asn23, Asp46, and Gly68. A pronounced conformational change of Asp46 in the central loop appears to facilitate the protein-calixarene interface. The Asp46 side chain rotates ∼90° from the usual conformation (a hydrogen bond with the Gly24-C^α^) to hydrogen bond with the amide NH of Gly68. This new conformation eliminates a potential steric clash with the bound **sclx**_**8**_ in crystal form III.

### NMR Analysis of RSL-**sclx**_**8**_ Complexation and the pH Trigger

**Figure 5:**
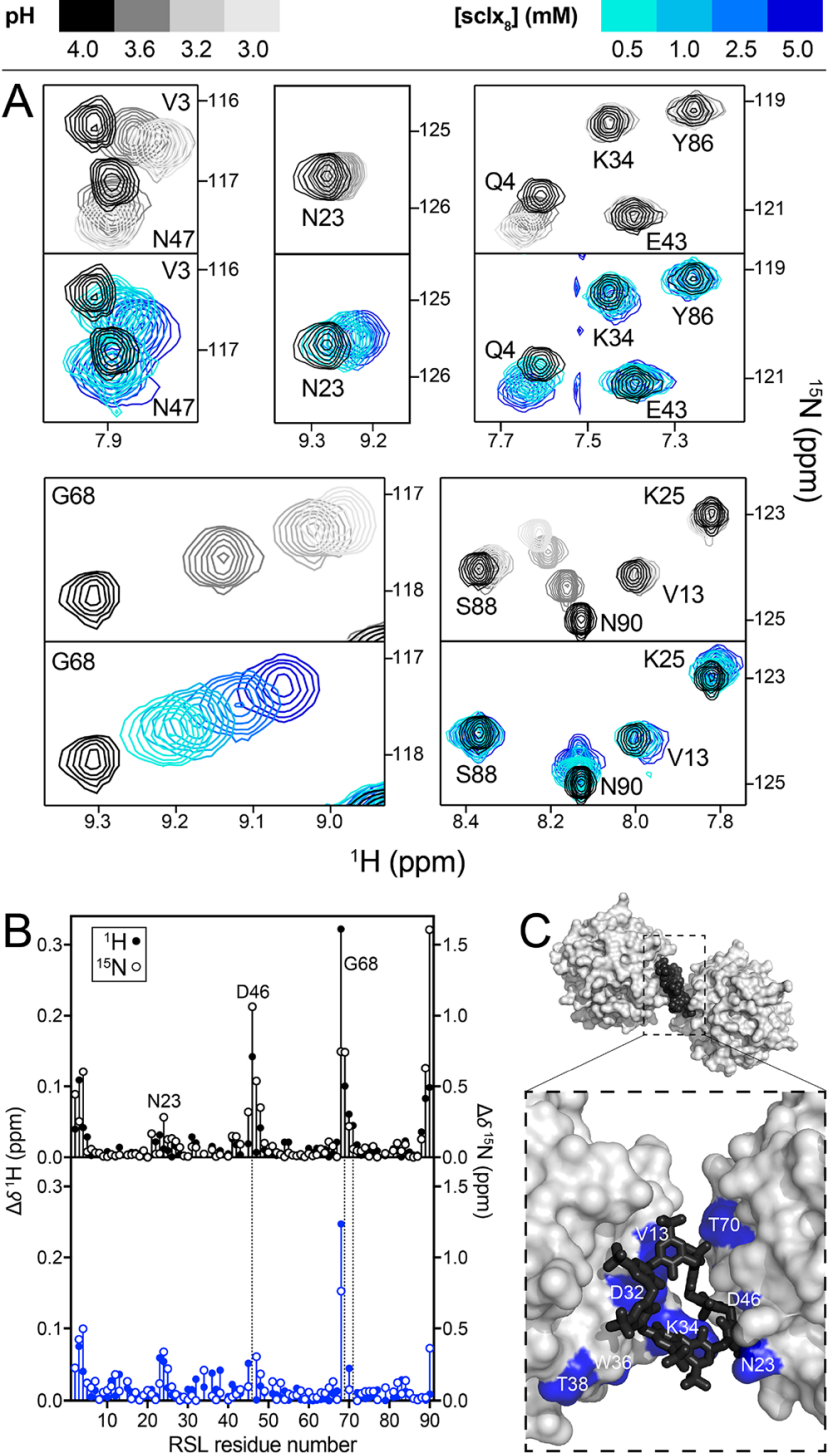
(A) Regions from overlaid ^1^H–^15^N HSQC spectra of RSL during pH (gray scale) or **sclx**_**8**_ (color scale) titrations. (B) Chemical shift perturbation plots of RSL backbone amides in response to pH adjustment (pH 4.0–3.0) or to the addition of 5 mM **sclx**_**8**_ (at pH 4.0). Dashed lines indicate unassigned resonances due to overlap. (C) Detail of the RSL-**sclx**_**8**_*P*3 assembly (Figure 2) showing RSL trimers in surface representation and bridging **sclx**_**8**_ as black spheres or sticks. Significant chemical shift perturbations at 5 mM **sclx**_**8**_ are highlighted blue.

**Figure 6:**
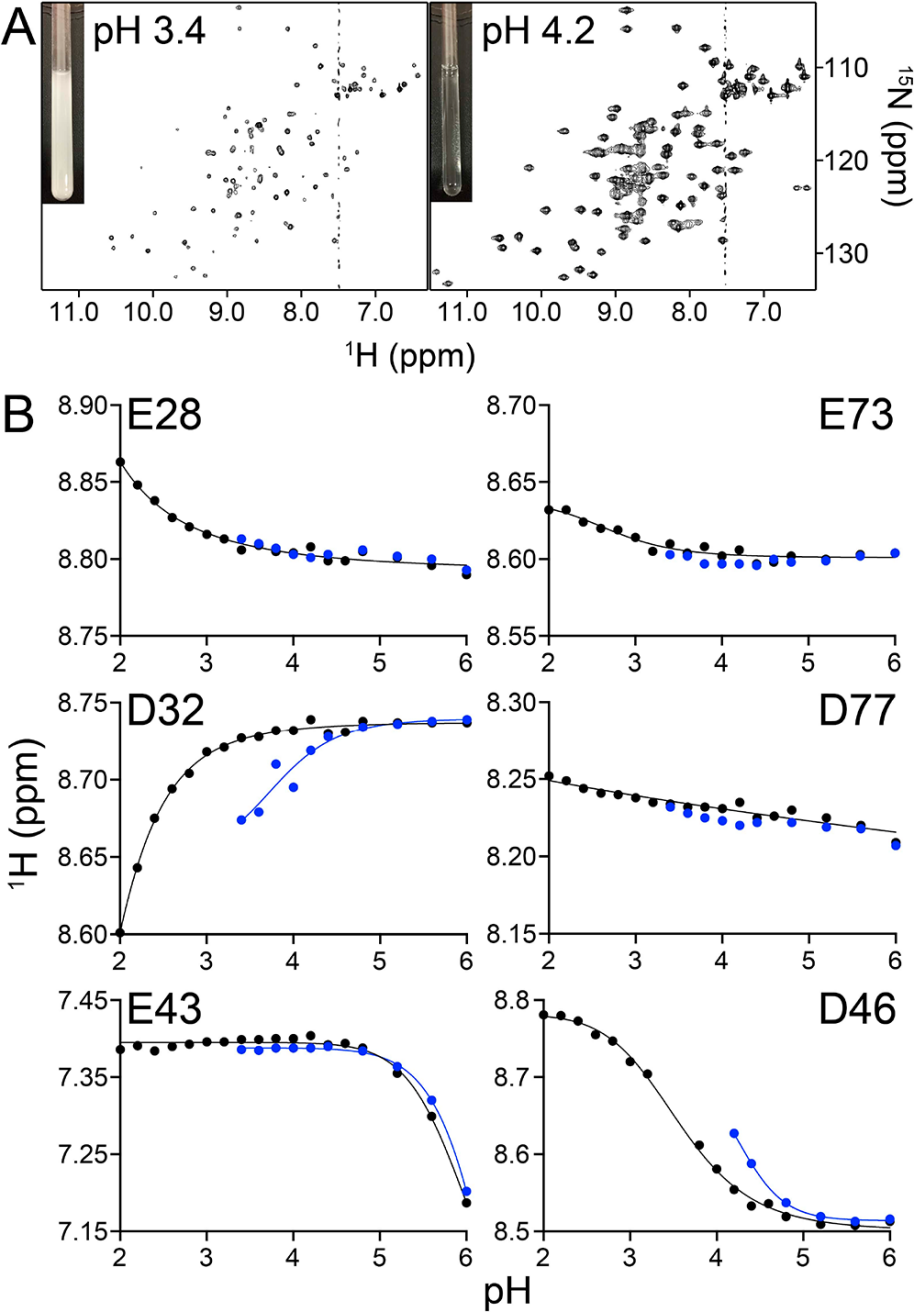
(A) RSL-**sclx**_**8**_ mixtures precipitated at pH ≤ 3.4, precluding NMR data collection. Precipitation was pH-reversible. (B) pH titration curves for the 6 acidic residues of RSL obtained by monitoring the ^1^H^N^ signal at 0 (black) or 5 mM (blue) **sclx**_**8**_.

The pH effects identified in the cocrystallization experiments were evident also from solution-state NMR experiments in 20 mM phosphate (or acetate) buffer and 50 mM NaCl. At pH 5.6, the ^1^H–^15^N HSQC spectrum of RSL was unchanged by titration with mM concentrations of **sclx**_**8**_, suggesting that RSL and **sclx**_**8**_ have negligible interactions under these conditions (Figure S9). However, at pH 4.0, specific RSL resonances were perturbed significantly by **sclx**_**8**_, with saturation occurring at ∼10 equiv **sclx**_**8**_ (Figures 5 and S9). Analysis of the chemical shift perturbations as a function of **sclx**_**8**_ concentration yielded hyperbolic binding curves (Figure S10) with binding affinity in the mM range. Mapping the significantly affected resonances onto crystal form III (*P*3) revealed binding patches consistent with the crystallographically defined protein-calixarene interfaces (Figure 5C). The occurrence of both binding sites (as per crystal form III) in the NMR experiments suggests transient sharing of bound **sclx**_**8**_ between two RSL trimers. This inference is corroborated by line broadening evidence. The average ^1^H^N^ line-widths of RSL increased by ∼8 Hz in the presence of **sclx**_**8**_ (Table S6), consistent with complexation and assembly in solution.^34,35^ Attempts to characterize RSL-**sclx**_**8**_ at lower pH values were thwarted by precipitation at pH ≤ 3.4 and severe signal loss in the HSQC spectrum (Figure 6A). These observations agreed with the results of the cocrystallization experiments (Figure 4A). Precipitation in the NMR sample was fully reversible. Increasing the sample pH to 4.2 resulted in complete dissolution of the precipitate (within seconds) and concomitant restoration of the spectrum.

**Figure 7:**
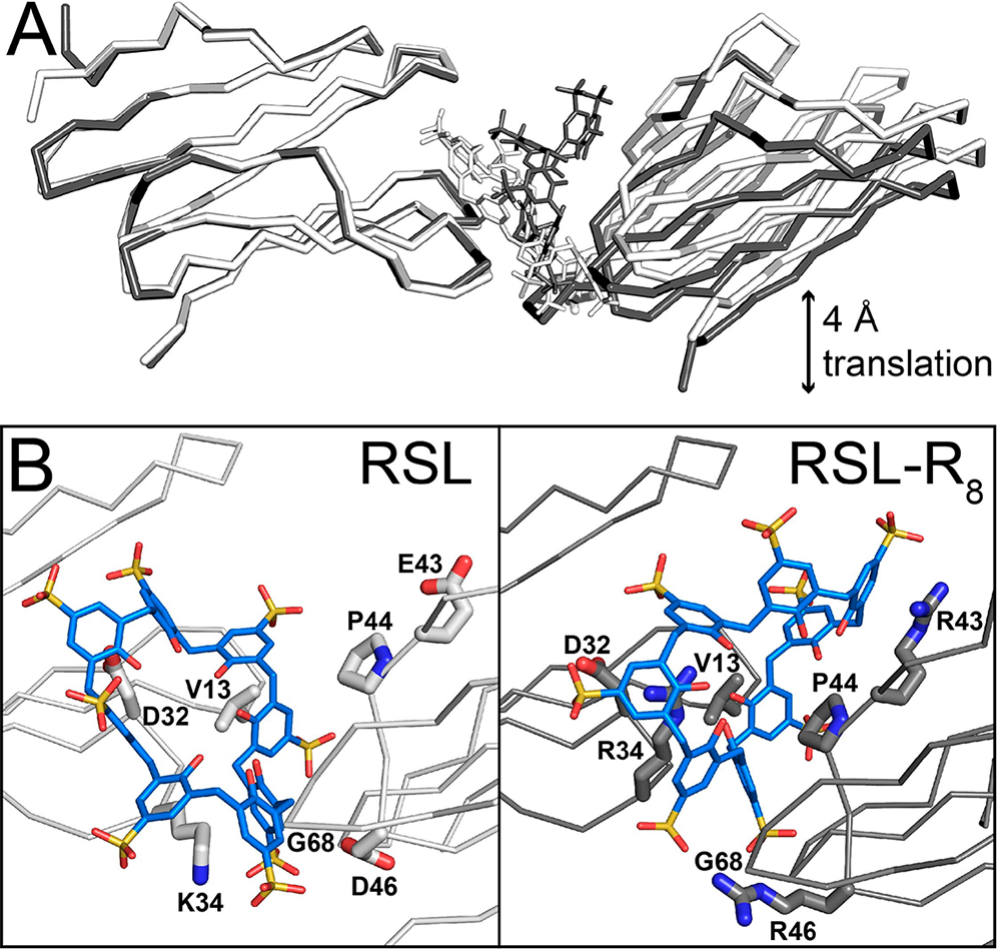
(A) Structural alignment (via chain A) of the main protein-sclx_8_-protein assembly in the *P*3 crystal forms of RSL (white) and RSL-R_8_ (gray). One of the proteins is translated by ∼4 Å in the RSL-R_8_ structure. (B) Detail of the protein-**sclx_8_**-protein interfaces with key side chains shown as sticks.

RSL-**sclx**_**8**_ complexation at low pH is due to the increased cationic character of the protein. In the course of the NMR experiments, we observed a striking similarity between the chemical shift perturbations induced by **sclx**_**8**_ and the perturbations produced by pH titration of the pure protein (Figure 5). The amide resonance of Gly68 shifted strongly, with the same direction and magnitude, due to a 1 pH unit change or to a 10-fold increase in **sclx**_**8**_ concentration. Similar effects occurred for N-terminal residues (Val3, Gln4) and Asn47. Both Asn47 and Gly68 are reporters on Asp46, the resonance of which occurs in a crowded region of the spectrum. Other resonances such as Val13, Lys25, and Lys34, implicated in **sclx**_**8**_ binding, were affected by the addition of **sclx**_**8**_ but not by changes in pH. Yet others were strongly affected by pH (e.g., C-terminal Asn90) but less so by **sclx**_**8**_. These effects were independent of the buffer, either phosphate or acetate, and suggest that calixarene binding involves protonation of RSL. Asp32 and Asp46 are the obvious candidates considering that they contribute to the protein-calixarene-protein interfaces in crystal form III (*P*3). Asp32 is buried completely by the calixarene while the solvent accessibility of Asp46 is unaffected, and both side chains undergo significant conformational changes (Figures 5C and 7B). In particular, the **sclx**_**8**_-directed assembly appears to be contingent on a structural rearrangement of Asp46, which flips to form a new hydrogen bond with Gly68.

Further evidence for a pH trigger for protein-calixarene assembly was provided by p*K*_a_ analysis of the acidic side chains in the absence and presence of **sclx**_**8**_ (Figures 6 and S11). The NMR-derived p*K*_*a*_ values for the acidic residues of RSL were in good agreement with the values calculated in PROPKA3.2 (Table S7).^69^ RSL has six acidic residues, including structurally equivalent pairs (Glu28/Glu73 and Asp32/Asp77) located in the homologous N- and C-terminal halves of the protein.^39,43^ The pH titration curves of Glu28/Glu73, which are completely buried (Table S7) and do not participate in calixarene complexation, were unaffected by the addition of 5 mM **sclx**_**8**_. Similarly, Glu43, which stacks coplanar with the Trp74 side chain, has a high p*K*_a_ value (5.9) that was unaffected by **sclx**_**8**_. In the case of Asp32, the p*K*_a_ was elevated by 2 pH units to 3.7 in the presence of **sclx**_**8**_. However, **sclx**_**8**_ had no effect on the structurally equivalent but nonbinding Asp77 (Figure 6B and Table S7). The p*K*_a_ of Asp46 was similarly elevated, but a value was not determined directly due to spectral overlap. Data for Asn23 and Gly68, which flank Asp46, yield a p*K*_a_ of ∼4.2 (Figure S11). The significantly increased p*K*_a_ values of Asp32 and Asp46 are consistent with protonation upon **sclx**_**8**_ binding at pH ∼ 4. PROPKA3.2 calculations yielded similar p*K*_a_ elevations due to **sclx**_**8**_ (Table S7). The aromatic shielding and the negative potential of the calixarene favor the protonated state of these side chains. Host–guest p*K*_a_ elevation is well-known in small molecule systems.^38,72−75^ Now, we show that such effects apply to proteins also,^76^ with consequences for macrocycle-mediated assembly. Considering pH effects, the charge-state of **sclx**_**8**_ must be considered also. While the sulfonic acids are always deprotonated in water, 4 of the phenolic groups have p*K*_a_ values ranging from ∼2 to ∼8.5, at 0.1 M ionic strength.^38^ At pH 4, **sclx**_**8**_ carries a formal net charge of −10 while the RSL trimer is +11, accounting for calixarene-induced protonation of Asp32 and Asp46. At pH 5.6, where binding does not occur, the formal charge of **sclx**_**8**_ is unchanged while RSL is +5 (Table S7).

### Versatility of *P*3 Framework and Effect of Arginine Enrichment

The facile fabrication of crystal form III (*P*3) makes it a prime candidate for crystal engineering. To this end, the *P*3 cocrystallization method (incubation of protein-**sclx**_**8**_ mixtures at pH 4.0 and 4 °C) was applied to several chemically modified or mutated RSL variants (Figures S6 and S7). The methylated protein (RSL*), bearing dimethylated lysines with increased affinity for **sclx**_**n**_,^77,78^ behaved in a manner similar to the native protein. Crystals grew within hours and were essentially identical to the native RSL-**sclx**_**8**_ structure (Tables 1 and S4, Figure S12). Mean isotropic temperature factor comparisons^57^ indicated a more rigid assembly for the dimethylated protein compared to the other variants (all of which were determined at similar resolution. Table S8). In contrast, the acetylated protein (RSL-Ac), bearing amide-terminated Lys side chains with negligible affinity for **sclx**_**n**_,^77^ did not cocrystallize. No localized precipitate was observed during the pH-adjustment of RSL-Ac and **sclx**_**8**_ mixtures, which remained soluble even at pH 2.0. The introduction of an additional Lys residue into RSL was in some cases compatible with the *P*3 assembly. Previously, we generated the double mutant N79K/T82Y named RSLex (Figures S1 and S2).^43^ These mutations introduce a C-terminal feature homologous to the macrocycle binding site at Lys34 in the N-terminal blade. Interestingly, up to 10 equiv **sclx**_**8**_ caused precipitation of RSLex, indicating that this protein was more prone to macrocycle-mediated assembly than RSL. At 15 equiv or higher, the mixture was soluble suggesting that the protein is encapsulated by the calixarene.^35^ Crystals grew within hours, and the structure was identical to the original *P*3 assembly (Table 1, Figure S12). The new potential binding site at Lys79 did not interact with the calixarene in the crystal, suggesting that additional features are required to switch on complexation. The other mutant tested, MK-RSL, contains a Met-Lys motif as the N-terminus. In crystal form III, **sclx**_**8**_ has peripheral interactions with the N-terminus, which is disordered even though it is cationic. Despite the additional Lys, MK-RSL remained soluble in the presence of **sclx**_**8**_ and growth of the crystal form III was switched off, possibly because of the N-terminal disorder or steric hindrance.

The arginine-enriched mutants RSL-R_6_ and RSL-R_8_ deviated distinctly from the behavior of the native protein, forming amorphous precipitates at ≥0.5 equiv **sclx**_**8**_ (Figure S7). Soluble mixtures of RSL-R_n_ were obtained only at 20 equiv **sclx**_**8**_. Similar to RSLex, these observations suggest that low equiv **sclx**_**8**_ induces aggregation due to the “molecular glue” effect, while high equiv **sclx**_**8**_ yields soluble samples as each protein is encapsulated by the calixarene.^35^ Attempts to characterize such assemblies by solution NMR were hindered by the high concentration of **sclx**_**8**_ required and the concomitant ionic strength effects. The pH-dependent precipitation characterized for native RSL occurred similarly for RSL-R_n_ (Figure S7). The precipitated mixtures at 10 equiv **sclx**_**8**_ and pH 4.0 could be solubilized by adjusting the pH to 4.6 in the case of RSL-R_6_. However, RSL-R_8_ remained partly precipitated even at pH 8.0 due to its high p*I*. While the RSL-**sclx**_**8**_ crystal form III grew within hours, the RSL-R_n_ mutants exhibited no crystal growth in this condition. Conventional vapor-diffusion experiments in the presence of ammonium sulfate yielded cocrystals. RSL-R_6_ and RSL-R_8_ were cocrystallized with **sclx**_**8**_ under near-identical conditions (Table 1) while RSL-R_6_ resulted in crystal form I (*P2*_1_3, *vide supra*) and RSL-R_8_ crystallized rapidly (∼3 h) to yield crystal form III (*P*3, Figures 7 and S12). This remarkable result supports the concept of pH- or charge-controlled protein-macrocycle assembly. The charge properties of RSL at pH 4 are preserved in RSL-R_8_, which is highly cationic across the pH range and thus amenable to calixarene-mediated assembly.

Although RSL-R_8_ yielded crystal form III like RSL, the unit cell had a shorter *c* axis (60 Å compared to 64 Å, Table 1). Congruently, one protein chain in the asymmetric unit was translated by ∼4 Å relative to the original structure (Figure 7A). Although the positions of RSL-R_8_ and RSL are similar in the two structures, the bridging calixarenes have substantially different conformations and binding sites. A more puckered **sclx**_**8**_ occurs bound to RSL-R_8_, compared to RSL-**sclx**_**8**_ (Figure S13). Interestingly, although similar protein-**sclx**_**8**_ interface areas were formed in both structures, the principal interface was wholly modified. In RSL, the principal interface centers on Val13 and Lys34 (*vide supra*). In RSL-R_8_, binding at Val13 and Arg34 remains crucial to the assembly but the interface is ∼2-fold smaller than the corresponding interface in the native protein. Instead, the principal binding site involves the new arginines, Arg43 and Arg46, as well as Pro44. Apparently, this binding site fulfils a similar function to the protonated Asn23/Asp46/Gly68 patch in the native protein but with a ∼2-fold larger surface area. Arg43 and Pro44^36^ dominate this interface with cation-π and CH-π bonds to the calixarene. The RSL-R_8_ structure also suggests why RSL-R_6_ did not crystallize in crystal form III (*P*3). The calixarene conformation supported by Arg43 in RSL-R_8_ cannot be maintained by Glu43 in RSL-R_6_.

## Discussion

Anionic calixarenes are well-established “glues” for the assembly and crystallization of cationic proteins such as cyt*c*,^34,35,37^ lysozyme,^33,78^ and PAF.^36^ Here, we have demonstrated the cocrystallization of “neutral” RSL with highly anionic **sclx**_**8**_. Three distinct crystal forms were obtained across a broad pH range (2.2–9.5) raising the possibility of general applications of calixarene-mediated protein assembly. The tightly packed crystal form I (*P*2_1_3) grew at pH 4.8–9.5 and required >1.5 M ammonium sulfate, consistent with the hydrophobic effect, rather than charge–charge interactions, as the dominant contributor to assembly. At pH ≤ 4.2, two types of porous frameworks were obtained. At this pH, RSL is cationic, which favors calixarene binding. NMR experiments suggest that calixarene complexation alters the cationic character of the protein by raising the p*K*_a_ values of two acidic residues (Asp32 and Asp46). **sclx**_**8**_ complexation may also modulate the protonation of Lys34. The disorder of this side chain in the *P*2_1_3 structures suggests that it is deprotonated (Figure 2).

**Figure 8:**
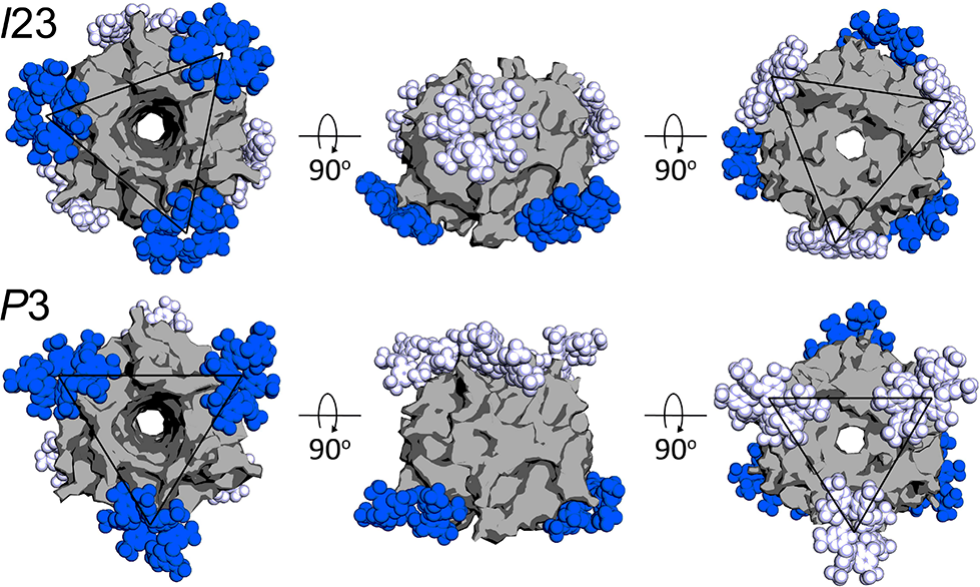
Calixarene encapsulation of RSL is evident in the building blocks of crystal forms II (*I*23) and III (*P*3). In each assembly, the RSL trimer (gray surface) is masked by six **sclx**_**8**_ molecules. The ligands are rendered in blue or white corresponding to the large and small interfaces, respectively. Black triangles indicate *C*_3_ planes that connect symmetry-related calixarenes.

Crystal forms II and III, are highly porous frameworks with pore diameters of ∼4 and ∼3 nm, respectively (Figure 1 and Table 2). The most porous assembly (form II, *I*23) was obtained at pH 2.2–4.0 and <1 M ammonium sulfate. This framework is built from **sclx**_**8**_ dimers and RSL trimers acting as nodes. Each node is a protein coated with six calixarenes in an octahedral arrangement (Figure 8). The resulting *primitive cubic* lattice has no protein–protein contacts and relies instead on protein-calixarene and calixarene-calixarene contacts (Figure 1). Protein assembly mediated by macrocycle dimers has been observed previously with porphyrins^44^ and calixarenes.^34^ For example, phosphonato-calix[6]arene forms a dimeric disc to mediate a porous assembly of cyt*c* (PDB 5LYC).^34^ Similar to crystal form II (*I*23), form III (*P*3) involves RSL trimers bridged together by six **sclx**_**8**_ ligands arranged in triads on two *C*_3_ planes (Figure 8). However, the positioning of the calixarene triads on opposite ends of the trimer disables the possibility of cubic assembly. Also like the *I*23 case, the *P*3 framework assembles only in acidic conditions (pH ≤ 4.0). Such pH-triggered assembly is an exciting new aspect of macrocycle-directed protein assembly. pH-dependent cationization as a driver of protein-macrocycle framework assembly was corroborated by the results with RSL-R_8_. This permanently cationic protein also yielded the *P*3 framework but under mildly alkaline conditions (Figures 7 and S12) where arginine side chains remain protonated.

Depending on the conditions, RSL-**sclx**_**8**_ mixtures remain soluble or form crystals ranging from microcrystalline precipitates to millimeter-scale crystals. For example, RSL and 10 equiv **sclx**_**8**_ yields a microcrystalline precipitate at pH ≤ 3.4 and room temperature (Figure S7). The precipitate is dissolved by raising the pH to >4.2 or by increasing the **sclx**_**8**_ concentration to 20 equiv (Figures 4A and S8). The effect of increasing pH is explained simply by the less cationic RSL having lower affinity for the calixarene. The interruption of precipitation at 20 equiv **sclx**_**8**_ suggests calixarene-coating or encapsulation of the protein.^35^ At low equiv, the calixarene bridges two or more proteins resulting in aggregation. At high equiv **sclx**_**8**_, each protein binding site is masked by calixarene and the “glue” activity is switched off as the interaction between protein-calixarene particles switches from attractive to repulsive. Both the *I*23 and the *P*3 frameworks, with significant macrocycle-masking of the symmetric protein building block (Figure 8), support the assumption of encapsulation^35^ in solution. This behavior is analogous to the reentrant condensation phase behavior of proteins in the presence of multivalent counterions.^51,52^ Protein encapsulation is currently a topic of intense development^35,50,79,80^ and macrocycle-masking may be a simple approach to tackle this challenge.

The precipitation of RSL-R_6_, RSL-R_8_, and RSLex operated via similar condensed regimes, but unlike RSL, the variants formed amorphous precipitates at pH 4.0 (Figure S7). Apparently, arginine-enrichment and the increased p*I* leads to enhanced calixarene binding and rapid precipitation. RSL-R_6_ and RSLex required pH 4.6 to switch off precipitation while the most cationic variant, RSL-R_8_, precipitated even at pH 8.0. Lysine to arginine mutagenesis tends to reduce protein solubility, a potential contributing factor in the precipitation of the arginine-enriched variants.^81^ At pH 4.0 and 10 equiv **sclx**_**8**_, the lysine-enriched variant RSLex displayed the same precipitation behavior as the Arg-rich variants, suggesting that the assembly of all three variants was a consequence of higher **sclx**_**8**_ affinity rather than reduced protein solubility. Unlike RSLex, soluble mixtures of RSL-R_6_ and RSL-R_8_ at high equiv **sclx**_**8**_ and low ionic strength yielded no cocrystals. The versatility of guanidinium groups at protein–protein and protein–ligand interfaces is well-established.^53,82−84^ We reason that the “stickiness” of the Arg-rich variants toward **sclx**_**8**_ yielded highly encapsulated protein-calixarene particles incapable of self-association. This hypothesis is supported by cocrystallization of RSL-R_6_ and RSL-R_8_ occurring only at high salt concentration wherein counterions compete for **sclx**_**8**_ complexation and lower its affinity for the protein surface.

With considering the importance of protein frameworks to both basic science and biotechnology, the availability of facile fabrication strategies is essential to stimulate progress. Vastly different strategies are currently in development and applicable in distinct settings. Significant progress has been achieved by harnessing naturally occurring frameworks such as protein cages,^6,15^ capsids,^8,46^ and crystals.^11,14^ For example, the supramolecular assembly of ferritin cages can be directed by metal-coordination sites.^15,24^ Protein crystals that grow naturally inside living cells have been used to capture cargo including dyes or enzymes.^11,14^ In a particularly striking example, a 32 kDa lipase was trapped within the pores of Cry3Aa crystals that grow in *Bacillus thuringiensis* cells.^14^ The strategies used to engineer frameworks include designed oligomerization,^28,47−49^ charge–charge interactions,^25−27^ and ligand-mediated assembly.^19,23,37,45^ The latter stands out as the most promising since (1) protein engineering is not required and (2) the assembly process can be regulated by the ligand concentration and/or the presence of inhibitors.^35,37^ Custom-made bivalent ligands comprising a sugar and rhodamine dye have been used to generate porous frameworks and other assemblies of concanavalin A.^19,23^ With biotech applications in mind, inexpensive, highly water-soluble and nontoxic ligands are desirable. Anionic calixarenes fit these requirements and there is increasing evidence of their utility for framework assembly.^34−37^ Here, we demonstrated a pH-triggered framework fabrication of a “neutral” protein with **sclx**_**8**_, in the presence or absence of a precipitant. The pH dependence affords a second level of control over assembly. Finally, the host–guest interactions of **sclx**_**8**_(29−32) confers the crystal with additional binding sites.^37^ The different pore dimensions in the frameworks (Table 2) along with the varying solvent accessibilities of the calixarene (∼35, 45 and 55% solvent exposed in crystal forms I, II and III, respectively, Figure 1 and Table S5) result in materials with different ligand uptake capacities.

## Conclusions

Various sophisticated frameworks have been described to date, usually with the requirement for protein engineering or specific assembly inducing ligands.^19,23,25−27,47−49^ Sulfonato-calixarenes are commercially available: biocompatible ligands that can be combined with protein building blocks to yield frameworks in a manner reminiscent of MOF manufacture using off-the-shelf reagents.^12^ The data presented here greatly widen the scope for **sclx**_**n**_-assisted protein assembly and crystallization to include “neutral” target proteins. The primary advantage is facile pH-controlled framework fabrication. pH-induced cationization of the protein enhances calixarene complexation and results in porous assemblies. p*K*_a_ modulation, well-characterized in protein–ligand binding^76^ and in small molecule host–guest systems,^72−75^ is a contributing factor to macrocycle-mediated protein assembly.

Rapid, scalable production of protein-based frameworks is essential for their development as biomaterials and biologic alternatives to MOFs/COFs. While the *I*23 framework required days to grow, the *P*3 framework grew within hours in the absence of precipitant. This rapid fabrication of millimeter-scale crystals (Figure 4) under pH control and without the need for precipitants or specialized equipment is attractive for manufacturing processes, including the purification of therapeutic proteins.^85^ Furthermore, the controlled nucleation and the formation of microcrystalline samples within minutes has applications in serial crystallography.^86,87^ Complementing these valuable crystallization properties, the sulfur-rich **sclx**_**8**_ is a promising phasing agent (Figure 3).

While calixarene-directed assembly is easily applied to proteins in their native state,^34−37^ protein engineering can complement the supramolecular strategy. Here, the permanently cationic Arg-rich variant RSL-R_8_ resulted in the same *P*3 framework as the native protein at low pH. Finally, both the *I*23 and the *P*3 frameworks provide evidence of protein encapsulation by **sclx**_**8**_. These strategies for controlled protein assembly have potential applications in therapeutics and smart biomaterials. The opportunity for auxiliary host–guest chemistry within protein-macrocycle frameworks^37^ makes these materials prime candidates for the uptake of biomolecules such as enzyme substrates or other proteins. Such developments boost the application of biosupramolecular chemistry as a general strategy for protein framework fabrication.

## Supporting Information Available

Sequences and electrostatic surfaces of RSL variants, representative cocrystals and conditions, X-ray data collection and refinement statistics, crystal structure details, NMR data and p*K*_a_ analysis (PDF)
