# Supplementary material for: Facile Fabrication of Protein–Macrocycle Frameworks
Source: J Am Chem Soc. 2021 Jan 20;143(4):1896–907. doi: 10.1021/jacs.0c10697 (PMC8154523; doi:10.1021/jacs.0c10697)
Supplement: Supplementary file 1 — ja0c10697_si_001.pdf [file ja0c10697_si_001.pdf]

SUPPORTING INFORMATION

Facile Fabrication of Protein – Macrocycle Frameworks

Kiefer O. Ramberg,[a] Sylvain Engilberge,[a,b] Tomasz Skorek,[a] and Peter B. Crowley*,[a]

[a]School of Chemistry, National University of Ireland Galway, University Road, Galway, H91 TK33, Ireland.

[b]Swiss Light Source, Paul Scherrer Institut, Villigen PSI, 5232, Switzerland

*Correspondence to:

peter.crowley@nuigalway.ie

+353 91 49 24 80

Contents

**Figure S1**	Sequence alignment of RSL and variants	S3
**Figure S2**	Electrostatic surface representations of RSL and variants	S4
**Figure S3**	ESI^+ mass spectra of RSL-R_8 and MK-RSL	S5
**Table S1**	Predicted and measured masses from ESI^+ mass spectra	S5
**Figure S4**	Representative RSL-R_n and **sclx$_8$** co-crystals	S6
**Table S2**	$P2_13$, X-ray data collection, processing and refinement statistics	S7
**Table S3**	*I*23, X-ray data collection, processing and refinement statistics	S8
**Table S4**	*P*3, X-ray data collection, processing and refinement statistics	S9
**Figure S5**	The **sclx$_8$** dimer in the *I*23 crystal form	S10
**Figure S6**	Summary of the *P*3 crystallization experiments	S11
**Figure S7**	RSL and variants in the presence of 10 eq. **sclx$_8$** and varying pH	S12
**Figure S8**	RSL-**sclx$_8$** precipitate at pH 3.4 is solubilized by increasing **sclx$_8$** concentration	S13
**Table S5**	Surface areas of **sclx$_8$**-mediated interfaces	S14
**Figure S9**	^1H-^{15}N HSQC spectra of RSL-**sclx$_8$** titrations at pH 5.6 or 4.0	S15
**Figure S10**	Binding isotherms for RSL resonances during **sclx$_8$** titration at pH 4.0	S16
**Table S6**	$^1H^N$ line widths from HSQC spectra of RSL at 0 or 5 mM **sclx$_8$**	S17
**Figure S11**	pH titration curves for N23 and G68, reporters for D46	S18
**Table S7**	Measured pK_a values and net charge of RSL, based on NMR and PROPKA3	S19
**Figure S12**	Crystal packing in *P*3 frameworks of RSL and RSL-R_8	S20
**Table S8**	Temperature factors of the *P*3 co-crystal structures	S21
**Figure S13**	**sclx$_8$** conformations in the *P*3 co-crystal structures with RSL or RSL-R_8	S22

	10	20	30	40	50
**RSL**	SSVQTAATSW	GTVPSI**R**VYT	ANNG**K**IT**ER**C	W**D**G**K**GWYTGA	FN**E**PG**D**NVSV
**MK-RSL**	M**K**SVQTAATSW	GTVPSIRVYT	ANNGKITERC	WDGKGWYTGA	FNEPGDNVSV
**RSLex**	SSVQTAATSW	GTVPSIRVYT	ANNGKITERC	WDGKGWYTGA	FNEPGDNVSV
**RSL-R_6**	SSVQTAATSW	GTVPSIRVYT	ANNG**R**ITERC	WDG**R**GWYTGA	FNEPGDNVSV
**RSL-R_8**	SSVQTAATSW	GTVPSIRVYT	ANNG**R**ITERC	WDG**R**GWYTGA	FN**R**PG**R**NVSV

	60	70	80	90
**RSL**	TSWLVGSAI**H**	I**R**VYASTGTT	TT**E**WCW**D**GNG	WT**K**GAYTATN
**MK-RSL**	TSWLVGSAIH	IRVYASTGTT	TTEWCWDGNG	WTKGAYTATN
**RSLex**	TSWLVGSAIH	IRVYASTGTT	TTEWCWDG**K**G	WYKGAYTATN
**RSL-R_6**	TSWLVGSAIH	IRVYASTGTT	TTEWCWDGNG	WT**R**GAYTATN
**RSL-R_8**	TSWLVGSAIH	IRVYASTGTT	TTEWCWDGNG	WT**R**GAYTATN

Figure S1. Sequence alignment of RSL and the variants used in this study. In the RSL sequence, basic and acidic residues are highlighted blue and red, respectively. Residues mutated to Arg or Lys are in bold.

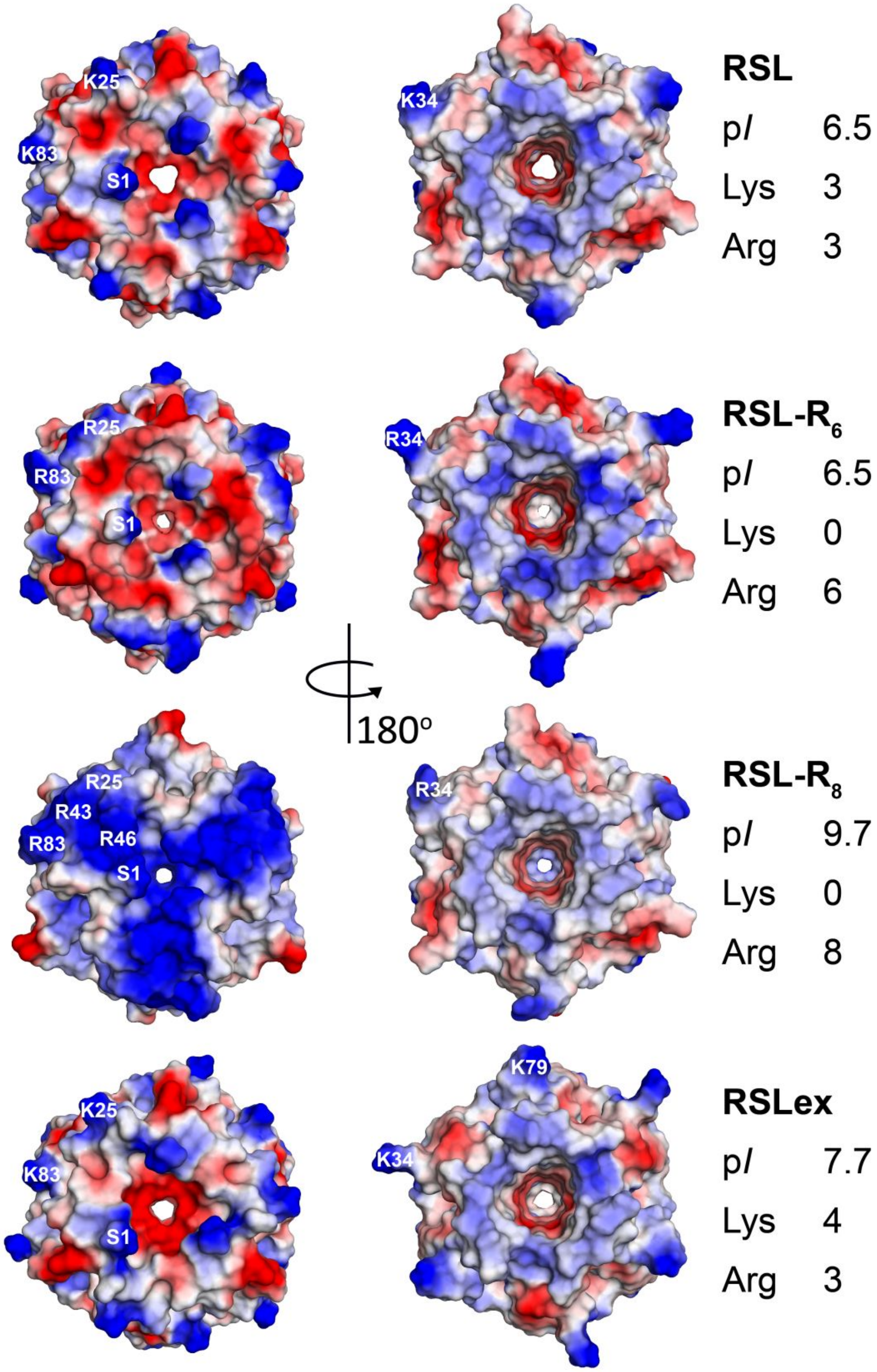

Figure S2. Electrostatic surface representations of RSL and variants. The calculated isoelectric point (p*I*) and the number of cationic residues per monomer are indicated. MK-RSL is not included as it has a disordered N-terminus.

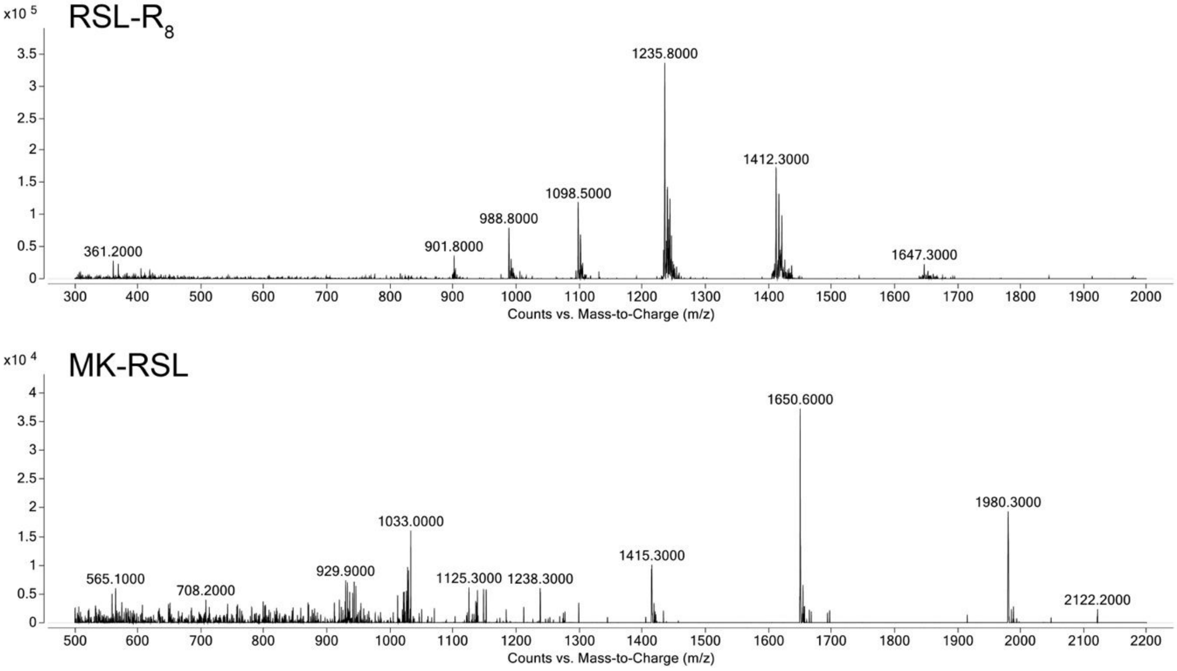

Figure S3. ESI⁺ mass spectra for RSL-R_8 and MK-RSL.

Table S1. Predicted and measured masses from ESI⁺ mass spectra (Figure S1).

		**RSL-R_8 monomer**	
**m/z**	**charge**	**Molecular Weight (Da)**	**Error (Da)**
1098.5	9+	9877.4	-0.8
1235.8	8+	9878.3	0.1
1412.3	7+	9879.0	0.8
Predicted MW (Da)		9878.8	
Deconvoluted MW (Da)		9878.3	
Standard deviation (Da)		0.8	

		**MK-RSL monomer**	
**m/z**	**charge**	**Molecular Weight (Da)**	**Error (Da)**
1415.3	9+	9900.0	2.0
1650.6	8+	9897.6	-0.5
1980.3	7+	9896.5	-1.6
Predicted MW (Da)		9899.9	
Deconvoluted MW (Da)		9898.0	
Standard deviation (Da)		1.8	

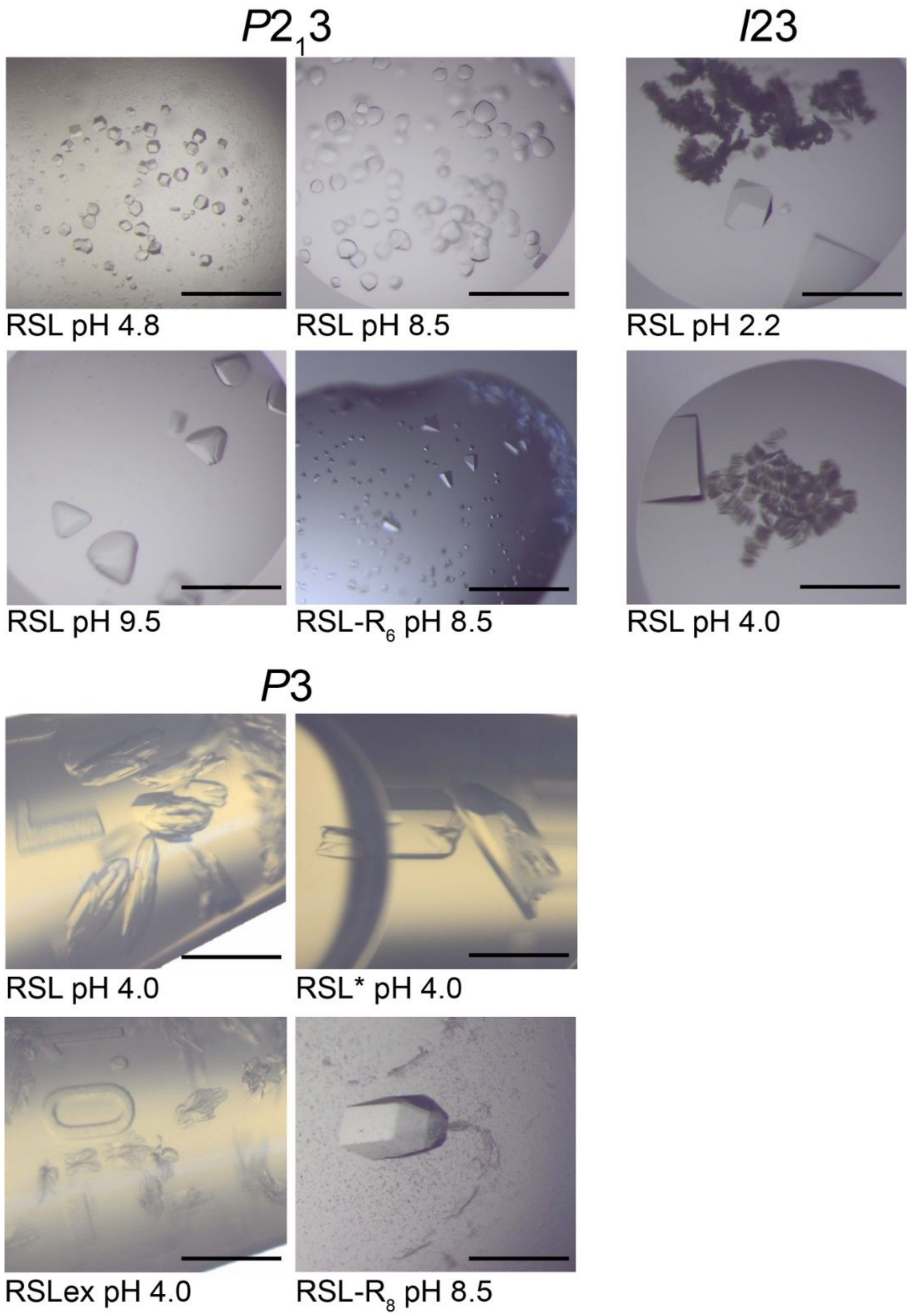

Figure S4. Representative co-crystals of RSL-R$_n$ and **sclx$_8$**. Scale bars are 200 µm. See Table 1 main text for conditions.

Table S2. X-ray data collection, processing and refinement statistics for the $P2_13$ crystal form.

			Data Collection		
**Light source**			SOLEIL, PROXIMA-2A		
**Wavelength (Å)**			0.98013		
**Structure**	RSL-$\mathbf{sclx_8}$ pH 4.8	RSL-$\mathbf{sclx_8}$ pH 6.8	RSL-$\mathbf{sclx_8}$ pH 8.5	RSL-R_6-$\mathbf{sclx_8}$ pH 8.5	RSL-$\mathbf{sclx_8}$ pH 9.5
**Space group**			$P2_13$		
**Cell constants (Å)**	64.05^3	63.83^3	63.94^3	63.82^3	63.87^3
**Resolution (Å)**	45.29-1.14 (1.15-1.14)	45.13-1.18 (1.20-1.18)	45.21-1.16 (1.18-1.16)	45.13-1.12 (1.14-1.12)	45.16-1.17 (1.19-1.17)
**# reflections**	1097412 (18080)	1042926 (27517)	184843 (47026)	1225434 (37714)	1068446 (22784)
**# unique reflections**	31827 (1364)	28303 (1395)	30746 (1527)	33221 (1632)	29919 (1474)
**Multiplicity**	34.5 (13.3)	36.8 (19.7)	38.5 (30.8)	36.9 (23.1)	35.7 (15.5)
**I/σ (I)**	25.1 (2.2)	19 (2.3)	25.2 (2.2)	19.3 (2.2)	35.8 (2.4)
**Completeness (%)**	98.7 (86.2)	100.0 (100.0)	100.0 (100.0)	100.0 (100.0)	100.0 (100.0)
$\boldsymbol{R_{meas}}^b$ **(%)**	9.6 (127.8)	12.8 (146.5)	7.7 (178.2)	16.8 (223.6)	4.8 (92.5)
$\boldsymbol{R_{pim}}^c$ **(%)**	1.6 (33.1)	2.1 (32.8)	1.2 (31.9)	2.7 (46.1)	0.8 (23.2)
$\mathbf{CC_{1/2}}$	100.0 (60.2)	99.8 (79.4)	99.9 (77.7)	99.8 (77.2)	100.0 (84.7)
**Solvent content (%)**	36	36	36	36	36
			Refinement		
$\boldsymbol{R_{work}}$	16.7	16.7	13.4	14.5	12.6
$\boldsymbol{R_{free}}$	19.1	17.7	15.4	16.8	15.0
**rmsd bonds (Å)**	0.004	0.006	0.006	0.007	0.007
**rmsd angles (°)**	0.835	0.892	0.991	0.932	0.982
			**# molecules in asymmetric unit**		
**Protein chains**	1	1	1	1	1
$\mathbf{sclx_8}$	1	1	1	1	1
**water**	119	132	129	142	173
**Ave. B-factor (Å^2)**	10.67	10.66	13.46	9.08	13.83
**Clashscore**	0.68	0.71	3.27	1.32	3.93
			**Ramachandran analysis,[d] % residues in**		
**favoured regions**	97.73	97.67	96.59	97.83	96.59
**allowed regions**	2.27	2.33	3.41	2.17	3.41
**PDB code**	6Z5X	6Z5W	6Z62	6Z60	6Z6Z

[a]Values in parentheses correspond to the highest resolution shell [b]$R_{meas} = \sum_{hkl} \sqrt{(n/n-1)} \sum_l |I_i(hkl) - \langle I(hkl) \rangle| / \sum_{hkl} \sum_i I_i(hkl)$; [c]$R_{pim} = \sum_{hkl} \sqrt{(1/n-1)} \sum^{n}_{i=1} |I_i(hkl) - \langle I(hkl) \rangle| / \sum_{hkl} \sum_i I_i(hkl)$; [d]Calculated in MolProbity.

Table S3. X-ray data collection, processing and refinement statistics for the *I*23 crystal form.

Data Collection			
**Light source**	SOLEIL, PROXIMA-2A	SLS, X06DA	SLS, X06DA
**Wavelength (Å)**	0.98013	0.97625	2.07505
**Structure**	RSL-**sclx$_8$** pH 2.2	RSL-**sclx$_8$** pH 4.0	RSL-**sclx$_8$** pH 4.0 – phasing
**Space group**		*I*23	
**Cell constants (Å)**	103.61^3	103.80^3	103.47^3
**Resolution (Å)**	73.26-1.6 (1.63-1.6)	42.37-1.28 (1.3-1.28)	51.73-2.04 (2.07-2.04)
**# reflections**	845994 (16845)	1910389 (88402)	333778 (291)
**# unique reflections**	24491 (1173)	48052 (2392)	11307 (238)
**Multiplicity**	34.5 (14.4)	39.8 (37.0)	29.5 (1.2)
**I/σ (I)**	26.4 (2.3)	29.9 (2.2)	72 (8.9)
**Completeness (%)**	99.8 (98.5)	100.0 (100.0)	95.3 (41.7)
**R_{meas}[b] (%)**	9.1 (93.1)	8.2 (21.0)	4.6 (8.6)
**R_{pim}[c] (%)**	1.5 (24.3)	1.8 (34.5)	0.8 (6.0)
**$CC_{1/2}$**	100.0 (85.7)	100.0 (77.4)	100.0 (98.7)
**|DANO|/sd(DANO)**	-	-	2.369 (0.914)
**Solvent content (%)**	66	66	66
Refinement			
**R_{work}**	13.9	15.5	
**R_{free}**	16.1	17.3	
**rmsd bonds (Å)**	0.007	0.005	
**rmsd angles (°)**	0.892	0.780	
**# molecules in asymmetric unit**			
**Protein chains**	1	1	
**sclx$_8$**	2	2	
**water**	349	203	
**Ave. B-factor (Å^2)**	19.76	22.0	
**Clashscore**	0.61	0.6	
**Ramachandran analysis,[d] % residues in**			
**favoured regions**	95.45	95.45	
**allowed regions**	4.55	4.55	
**PDB code**	6Z5G	6Z5M	

[a]Values in parentheses correspond to the highest resolution shell [b]$R_{meas} = \sum_{hkl} \sqrt{(n/n-1)} \sum_{I} |I_i(hkl) - \langle I(hkl) \rangle| / \sum_{hkl} \sum_i I_i(hkl)$; [c]$R_{pim} = \sum_{hkl} \sqrt{(1/n-1)} \sum^{n}_{i=1} |I_i(hkl) - \langle I(hkl) \rangle| / \sum_{hkl} \sum_i I_i(hkl)$; [d]Calculated in MolProbity.

Table S4. X-ray data collection, processing and refinement statistics for the *P*3 crystal form.

Data Collection				
**Light source**	SOLEIL, PROXIMA-2A			
**Wavelength (Å)**	0.98013			
**Structure**	RSL-**sclx**$_8$ pH 4.0	RSL*-**sclx**$_8$ pH 4.0	RSLex-**sclx**$_8$ pH 4.0	RSL-R_8-**sclx**$_8$ pH 8.5
**Space group**	*P*3			
**Cell constants (Å)**	59.85, 59.85, 64.63	59.48, 59.48, 64.72	59.68, 59.68, 64.28	60.06, 60.06, 59.60
**Resolution (Å)**	51.83-1.29 (1.32-1.29)	64.72-1.26 (1.28-1.26)	27.29-1.45 (1.48-1.45)	59.60-1.42 (1.44-1.42)
**# reflections**	661854 (30662)	695123 (27863)	463029 (22001)	474746 (24397)
**# unique reflections**	64914 (3257)	68362 (3377)	45237 (2252)	45137 (2253)
**Multiplicity**	10.2 (9.4)	10.2 (8.3)	10.2 (9.8)	10.5 (10.8)
**I/σ (I)**	13.5 (2.3)	16.4 (2.2)	15.7 (2.2)	23.8 (2.1)
**Completeness (%)**	100.0 (100.0)	99.0 (97.1)	100.0 (100.0)	99.0 (98.0)
R_{meas}[b] **(%)**	7.8 (68.9)	6.1 (73.2)	6.8 (83.5)	4.2 (109.2)
R_{pim}[c] **(%)**	2.4 (22.2)	2.8 (35.1)	3.0 (37.7)	1.3 (32.9)
$CC_{1/2}$	99.9 (95.1)	100.0 (92.4)	100.0 (91.5)	100.0 (82.6)
**Solvent content (%)**	59	59	59	59
Refinement				
R_{work}	12.1	14.5	14.5	10.8
R_{free}	15.3	16.2	16.8	14.2
**rmsd bonds (Å)**	0.006	0.004	0.005	0.006
**rmsd angles (°)**	0.903	0.796	0.758	0.973
**# molecules in asymmetric unit**				
**Protein chains**	2	2	2	2
**sclx**$_8$	2	2	2	2
**water**	578	369	352	448
**Ave. B-factor (Å^2)**	24.41	14.19	18.56	21.06
**Clashscore**	2.60	1.02	1.70	1.34
**Ramachandran analysis,[d] % residues in**				
**favoured regions**	96.59	96.59	97.73	97.16
**allowed regions**	3.41	3.41	2.27	2.84
**PDB code**	6Z5Q	7ALF	7ALG	6Z5P

[a]Values in parentheses correspond to the highest resolution shell [b]$R_{meas} = \sum_{hkl} \sqrt{n/(n-1)} \sum_{i} |I_i(hkl) - \langle I(hkl) \rangle| / \sum_{hkl} \sum_i I_i(hkl)$; [c]$R_{pim} = \sum_{hkl} \sqrt{1/(n-1)} \sum^{n}_{i=1} |I_i(hkl) - \langle I(hkl) \rangle| / \sum_{hkl} \sum_i I_i(hkl)$; [d]Calculated in MolProbity.

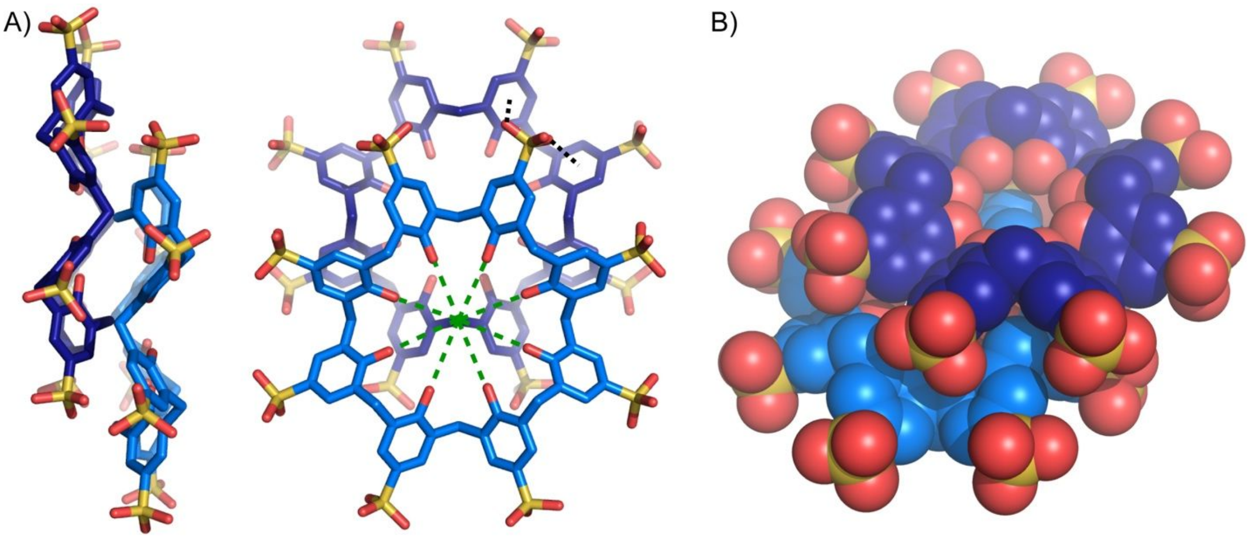

Figure S5. The *I*23 crystal form contains a symmetric **sclx$_8$** dimer shown in **(A)** sticks and **(B)** spacefill. Green dashed lines indicate van der Waals contacts (3.8-4.2 Å) between the phenol oxygens of one calixarene and a methylene bridge of the other calixarene. The black dashed lines indicate anion-π bonds between a sulfonate of one calixarene and a "calix[2]" motif of the other calixarene. The average $–SO_3^-$···centroid distance in the four equivalent sites is 3.8 Å.

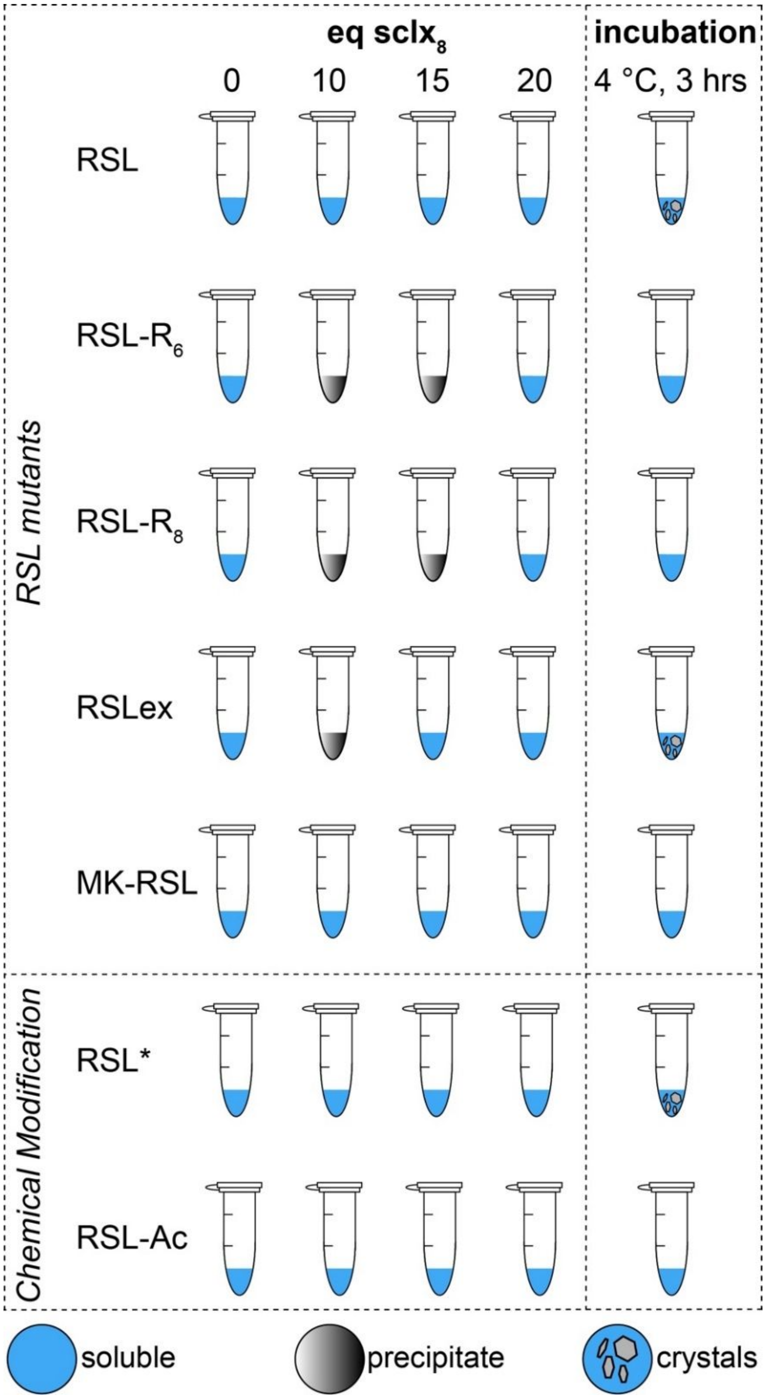

Figure S6. Summary of the *P*3 crystallization experiments in 20 mM acetate (or phosphate) buffer, 50 mM NaCl, at pH 4.0. Mixtures were prepared at room temperature and incubated at 4° C. The buffer pH was unaffected by temperature. During preparation, at room temperature, localised precipitation occurred as the sample pH was lowered. This precipitate dissolved immediately with mixing. Note that pure RSL is soluble at pH 2.0.

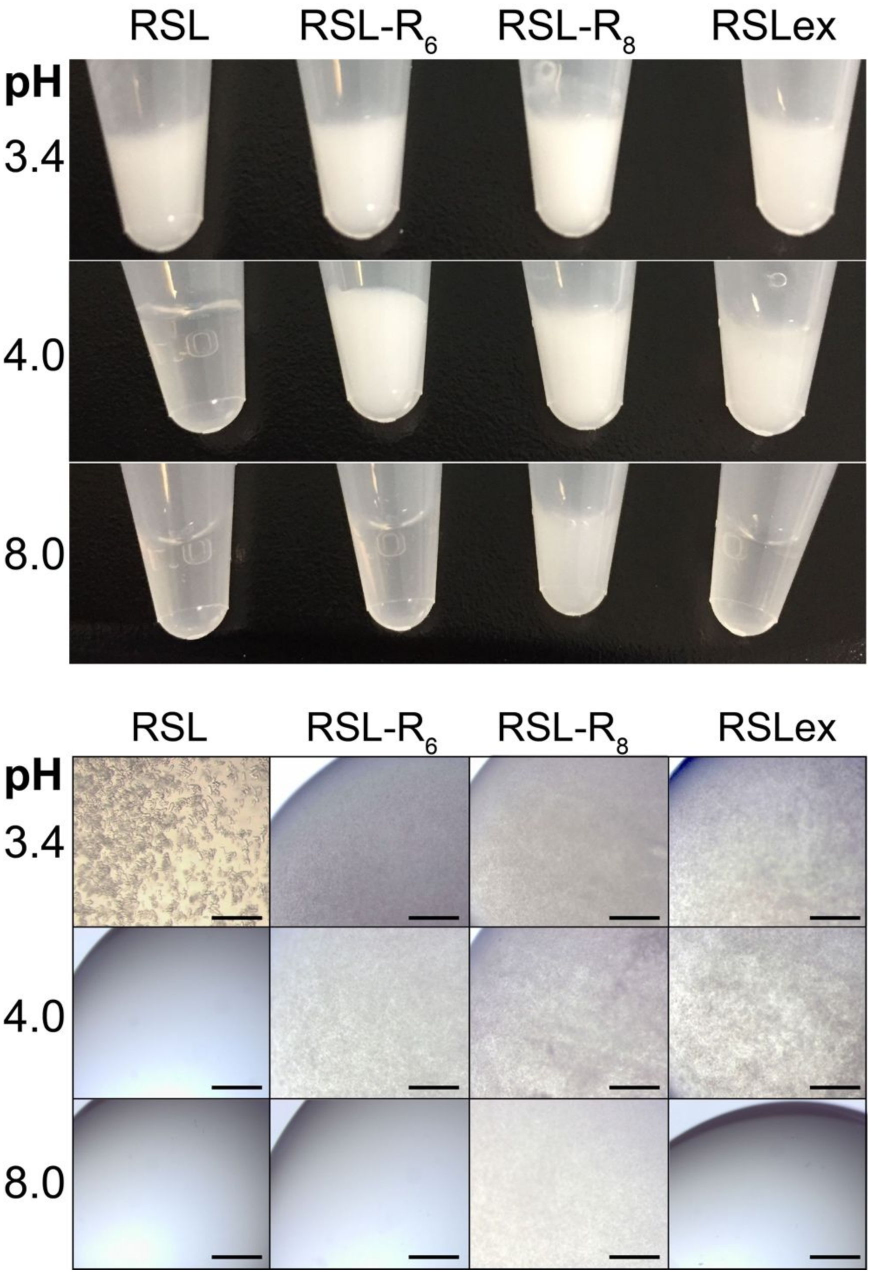

Figure S7. Room temperature precipitation tests of RSL and variants in the presence of 10 eq. **sclx$_8$** at varying pH in 20 mM phosphate and 50 mM NaCl. Similar results were obtained in acetate buffer. Lower panel, microscope images of the samples. Note the microcrystalline precipitate for RSL- **sclx$_8$** at pH 3.4. Precipitates were diluted 2-fold with buffer. Scale bars are 100 µm.

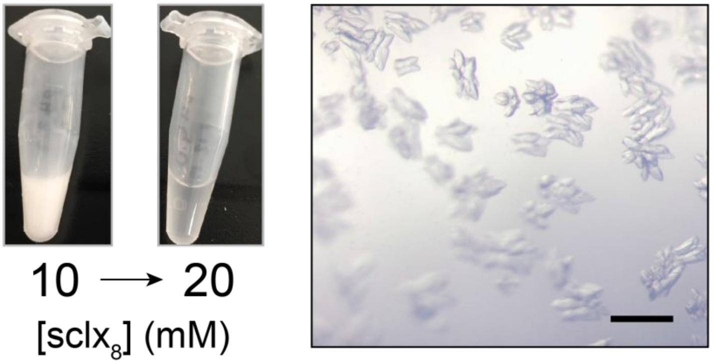

Figure S8. A mixture of 1 mM RSL and 10 mM **sclx$_8$** in 20 mM acetate and 50 mM NaCl precipitates at pH ≤ 3.4, room temperature. The precipitate dissolves upon increasing [**sclx$_8$**] to 20 mM. Incubation at 4° C for 3 hours yielded ample nucleation. Scale bar is 200 µm.

Table S5. Surface areas of **sclx$_8$**-mediated interfaces, determined in PISA.[21]

**Interface***	**Interface Area (Å^2)**		
	***P*2$_1$3**	***I*23[a]**	***P3* (RSL)**
L-P	1025	430	675
L-S	555	680	875
L-L	0	480	0

*L; ligand - **sclx$_8$**, P; protein - RSL, S; solvent - H_2O

[a]Average values for one **sclx$_8$**

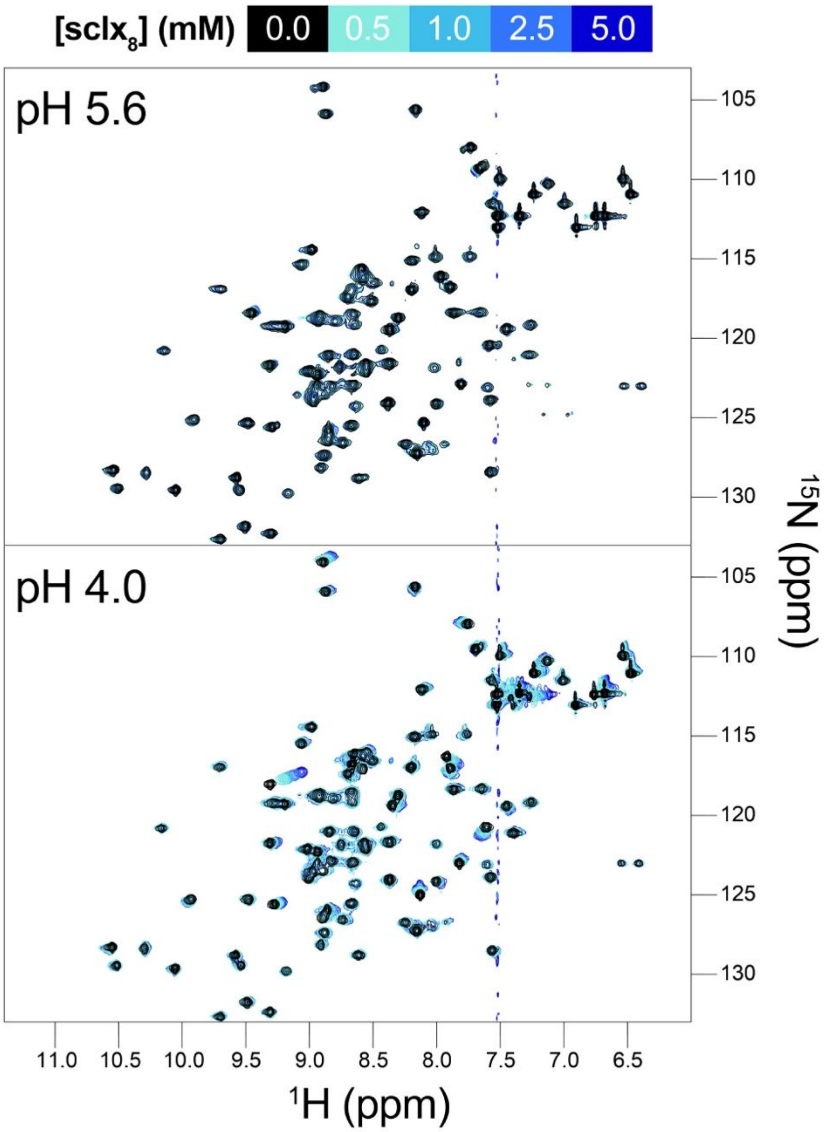

Figure S9. Overlaid ^{1}H-^{15}N HSQC spectra of 1 mM RSL and **sclx$_8$** (colour scale) at pH 5.6 or 4.0. The signal at ~7.5 ppm is due to **sclx$_8$**.

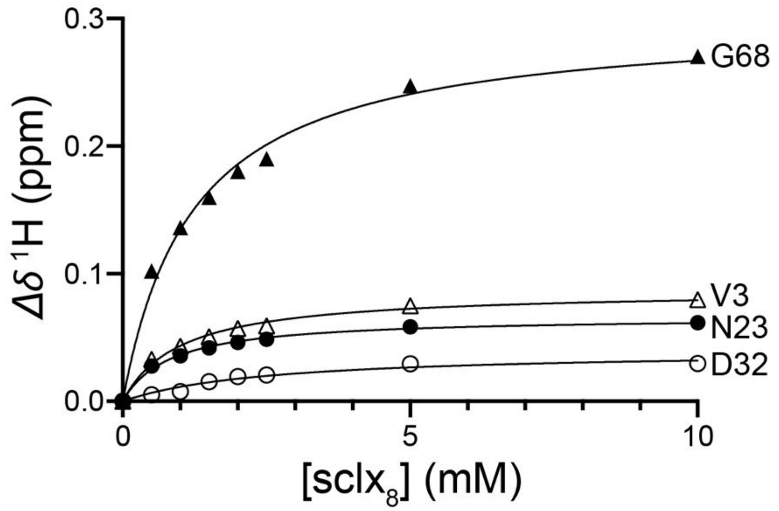

Figure S10. Hyperbolic binding isotherms for RSL resonances during titration with **$sclx_8$** at pH 4.0.

Table S6. $^1H^N$ line widths from HSQC spectra of RSL at 0 or 5 mM **sclx$_8$** and pH 4.0.

	**$^1H^N$ line-width (Hz)**			**$^1H^N$ line-width (Hz)**	
**Residue**	0 mM **sclx$_8$**	5 mM **sclx$_8$**	**Residue**	0 mM **sclx$_8$**	5 mM **sclx$_8$**
**S2**	15.8	23.3	**V48**	29.3	36.4
**V3**	19.3	25.1	**S49**	23.4	33.9
**Q4**	22.9	31.8	**S52**	19.0	25.7
**T5**	27.6	30.5	**W53**	28.7	37.0
**A6**	23.1	33.4	**V55**	27.8	35.7
**A7**	24.5	34.8	**G56**	21.8	34.2
**T8**	23.4	32.3	**S57**	20.3	27.7
**S9**	19.8	26.8	**A58**	21.0	26.1
**G11**	23.3	31.1	**I59**	25.7	35.6
**T12**	23.0	29.4	**H60**	35.3	38.8
**V13**	21.2	32.2	**Y64**	26.4	29.9
**S15**	25.9	34.2	**A65**	24.9	31.5
**R17**	25.5	35.4	**G68**	23.1	30.1
**Y19**	26.6	36.4	**T70**	19.9	26.8
**T20**	21.3	33.6	**T72**	27.1	35.5
**A21**	23.1	30.4	**E73**	27.0	33.5
**N22**	21.8	32.5	**W74**	23.1	37.5
**N23**	19.4	25.2	**C75**	25.3	37.6
**G24**	20.8	32.0	**D77**	27.3	35.6
**K25**	20.9	25.0	**D77**	27.3	35.6
**T27**	24.6	33.5	**N79**	24.3	29.6
**R29**	29.2	38.2	**W81**	27.4	33.8
**K34**	23.6	29.2	**K83**	19.1	30.3
**W36**	19.4	35.2	**G84**	27.0	33.6
**T38**	20.7	29.7	**Y86**	24.9	35.4
**F41**	28.6	32.6	**T87**	22.9	34.5
**N42**	25.2	29.8	**A88**	18.2	25.5
**E43**	26.4	32.8	**T89**	19.1	21.3
**G45**	19.6	29.2	**N90**	16.4	17.9
**N47**	20.6	29.2			
**Average line-width (Hz)**			0 mM **sclx$_8$** = 23.5 (±4)<br>5 mM **sclx$_8$** = 31.5 (±4)		

*Line-widths were measured in CCPNmr[8] for non-overlapping cross-peaks (~63 % of total). Resonances with line widths ≥ 40 Hz were considered outliers and were excluded from the analysis.

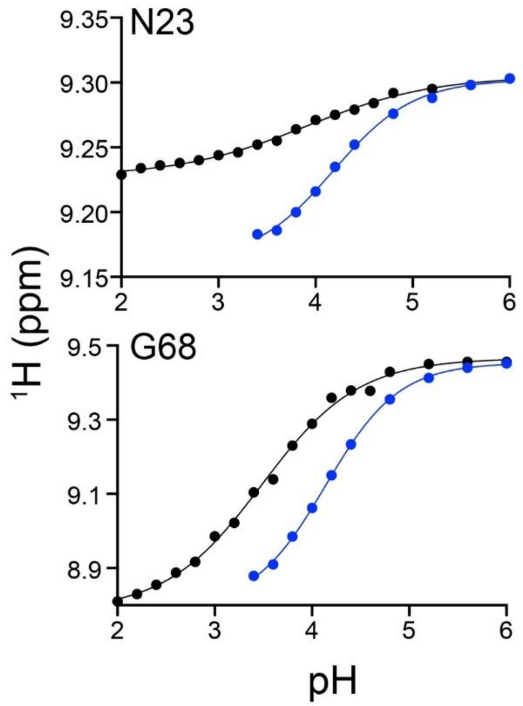

Figure S11. pH titration curves for N23 and G68, reporters for D46, in the presence of 0 (black) or 5 (blue) mM **sclx$_8$**. Precipitation at pH 3.4 in the presence of **sclx$_8$** precluded measurements at and below this pH.

Table S7. pK_a values of ionisable side chains in RSL and calculated formal charge.

Residue	pK_a calc.[a]		pK_a meas.[b]		Formal Charge		
	- $sclx_8$	+ $sclx_8$	- $sclx_8$	+ $sclx_8$	pH 5.6	pH 4.0	pH 4.0 + $sclx_8$
K25	10.4	10.3	> 6.0	> 6.0	+1	+1	+1
K34	10.5	10.1	> 6.0	> 6.0	+1	+1	+1
K83	10.4	10.3	> 6.0	> 6.0	+1	+1	+1
R17	13.1	13.1	> 6.0	> 6.0	+1	+1	+1
R29	12.3	12.3	> 6.0	> 6.0	+1	+1	+1
R62	13.0	13.0	> 6.0	> 6.0	+1	+1	+1
H60	7.0	7.0	> 6.0	> 6.0	+1	+1	+1
E28	4.2	4.5	< 2.0	< 2.0	-1	-1	-1
D32	2.4	3.2	1.7±0.02	3.7±0.01	-1	-1	-0.3
E43	5.8	6.1	5.9±0.01	6.2±0.01	-0.3	0	0
D46	3.6	4.2	3.5±0.01	4.2±0.01[c]	-1	-0.5	-0.2
E73	2.1	2.1	< 2.0	< 2.0	-1	-1	-1
D77	2.6	2.6	< 2.0	< 2.0	-1	-1	-1
				**Formal Net Charge (monomer)**	+1.7	+2.5	+3.7
				**Formal Net Charge (trimer)**	+5.1	+7.5	+11.1

[a]Values calculated in PROPKA3.2 using the *P*3 RSL-**$sclx_8$** structure (PDB 6Z5Q) with and without **$sclx_8$** coordinates.

[b]Values calculated from non-linear least-square fits of NMR data.

[c]Value based on reporter resonance Asn23 (Figure S11).

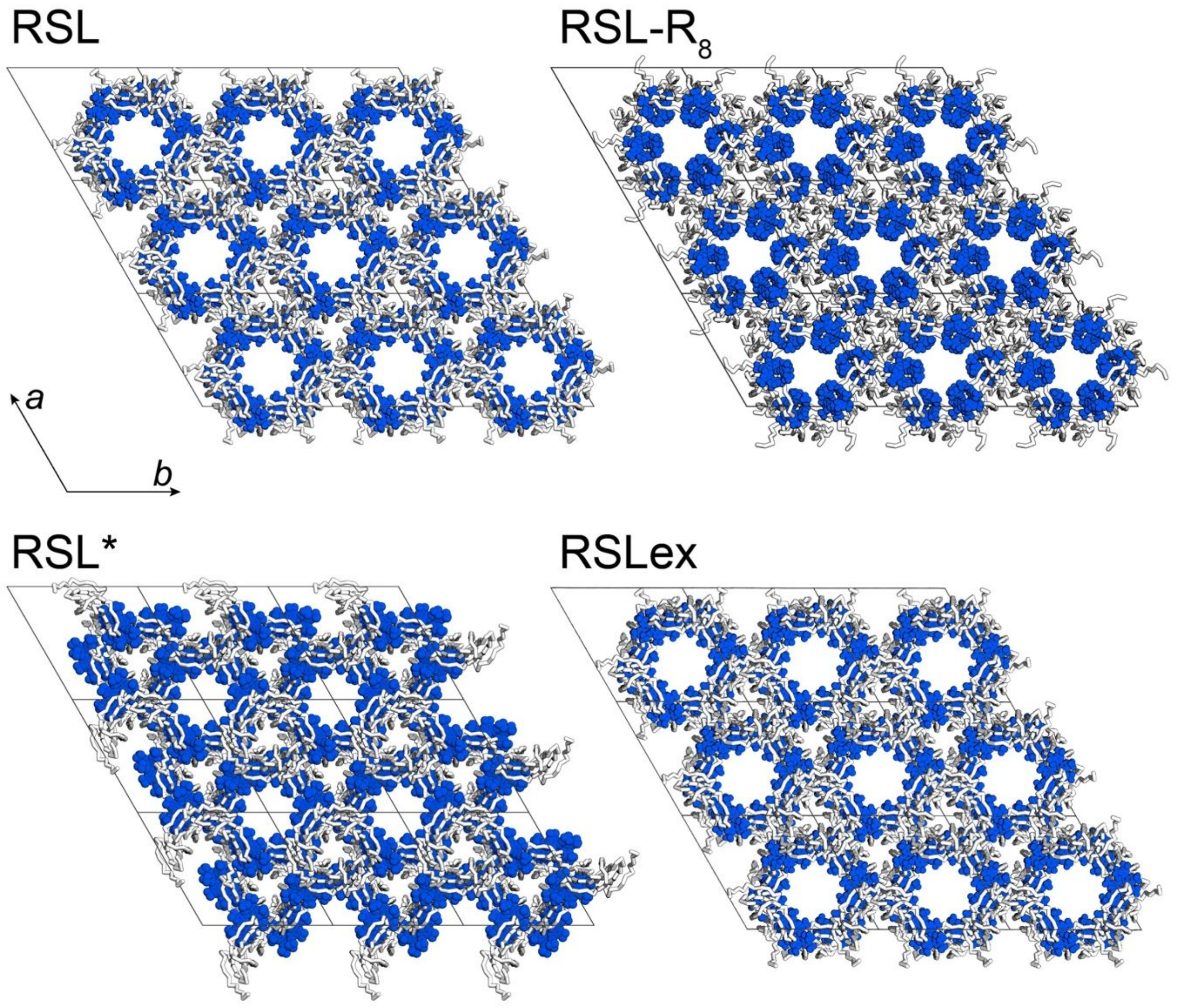

Figure S12. Crystal packing in the *P*3 frameworks of RSL and variants. Protein shown as grey ribbon and $\mathbf{sclx_8}$ as blue spheres. Unit cell axes are indicated with $a = b \approx 6$ nm.

Table S8. Mean all-atom isotropic temperature factors in *P*3 co-crystal structures.

Structure	Res (Å)	Chain	# Atoms	% Identity	Mean Isotropic Temperature factor (Å^2)	RMSD[a] (Å)
RSL-$\mathbf{sclx_8}$	1.3	A	681	100.0	16.5	-
		B	681		16.3	0.193
RSL*-$\mathbf{sclx_8}$	1.3	A	687	95.6	16.1	0.114
		B	687		15.9	0.339
RSLex-$\mathbf{sclx_8}$	1.5	A	687	97.8	20.6	0.117
		B	687		20.4	0.087
RSL-R_8-$\mathbf{sclx_8}$	1.4	A	692	94.4	27.1	0.825
		B	692		26.7	0.815

[a]Root-mean-square deviation of C^{α} atoms with respect to the reference structure.

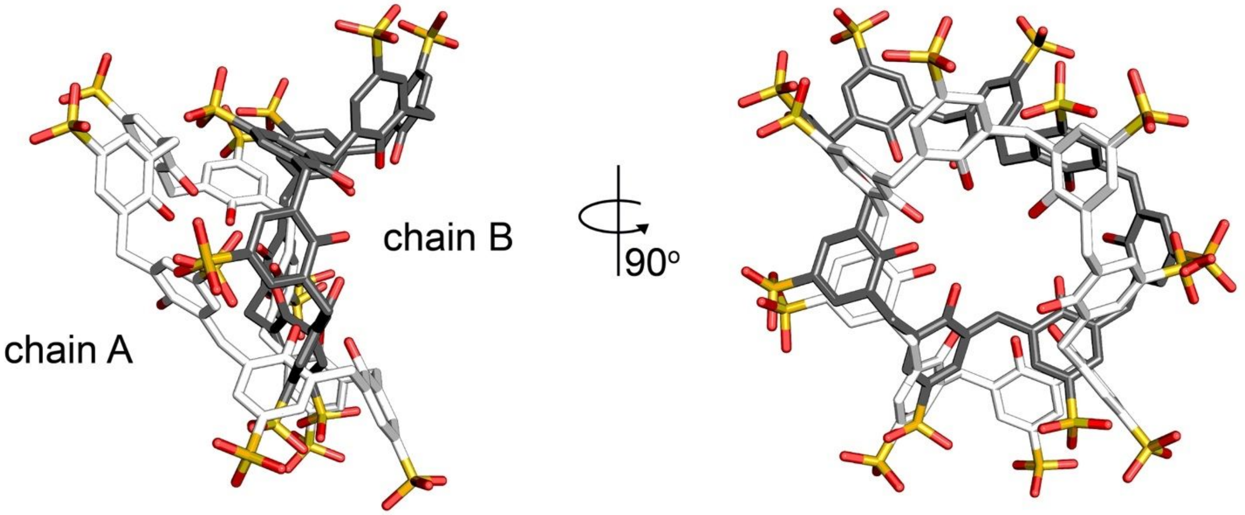

Figure S13. The conformations of **sclx₈** in the *P*3 co-crystal structures of **(A)** RSL and **(B)** RSL-R_8. Chain A and chain B refer to the approximate locations of the proteins (not shown) in the asymmetric units. Refer to main text Figure 7A.
